# Inflammatory, regulatory, and autophagy co-expression modules and hub genes underlie the peripheral immune response to human intracerebral hemorrhage

**DOI:** 10.1186/s12974-019-1433-4

**Published:** 2019-03-05

**Authors:** Marc Durocher, Bradley P. Ander, Glen Jickling, Farah Hamade, Heather Hull, Bodie Knepp, Da Zhi Liu, Xinhua Zhan, Anh Tran, Xiyuan Cheng, Kwan Ng, Alan Yee, Frank R. Sharp, Boryana Stamova

**Affiliations:** 1 Department of Neurology, University of California Davis School of Medicine, Sacramento, CA 95817 USA; 2 MIND Institute Biosciences Building, 2805 50th Street, Sacramento, CA 95817 USA

**Keywords:** Intracerebral hemorrhage, ICH, NRF2, Autophagy, Hematoma, Hematoma clearance, Src kinase inhibitors, Gene expression, Co-expression networks, Gene networks

## Abstract

**Background:**

Intracerebral hemorrhage (ICH) has a high morbidity and mortality. The peripheral immune system and cross-talk between peripheral blood and brain have been implicated in the ICH immune response. Thus, we delineated the gene networks associated with human ICH in the peripheral blood transcriptome. We also compared the differentially expressed genes in blood following ICH to a prior human study of perihematomal brain tissue.

**Methods:**

We performed peripheral blood whole-transcriptome analysis of ICH and matched vascular risk factor control subjects (*n* = 66). Gene co-expression network analysis identified groups of co-expressed genes (modules) associated with ICH and their most interconnected genes (hubs). Mixed-effects regression identified differentially expressed genes in ICH compared to controls.

**Results:**

Of seven ICH-associated modules, six were enriched with cell-specific genes: one neutrophil module, one neutrophil plus monocyte module, one T cell module, one Natural Killer cell module, and two erythroblast modules. The neutrophil/monocyte modules were enriched in inflammatory/immune pathways; the T cell module in T cell receptor signaling genes; and the Natural Killer cell module in genes regulating alternative splicing, epigenetic, and post-translational modifications. One erythroblast module was enriched in autophagy pathways implicated in experimental ICH, and NRF2 signaling implicated in hematoma clearance. Many hub genes or module members, such as IARS, mTOR, S1PR1, LCK, FYN, SKAP1, ITK, AMBRA1, NLRC4, IL6R, IL17RA, GAB2, MXD1, PIK3CD, NUMB, MAPK14, DDX24, EVL, TDP1, ATG3, WDFY3, GSK3B, STAT3, STX3, CSF3R, PIP4K2A, ANXA3, DGAT2, LRP10, FLOT2, ANK1, CR1, SLC4A1, and DYSF, have been implicated in neuroinflammation, cell death, transcriptional regulation, and some as experimental ICH therapeutic targets. Gene-level analysis revealed 1225 genes (FDR *p* < 0.05, fold-change > |1.2|) have altered expression in ICH in peripheral blood. There was significant overlap of the 1225 genes with dysregulated genes in human perihematomal brain tissue (*p* = 7 × 10^−3^). Overlapping genes were enriched for neutrophil-specific genes (*p* = 6.4 × 10^−08^) involved in interleukin, neuroinflammation, apoptosis, and PPAR signaling.

**Conclusions:**

This study delineates key processes underlying ICH pathophysiology, complements experimental ICH findings, and the hub genes significantly expand the list of novel ICH therapeutic targets. The overlap between blood and brain gene responses underscores the importance of examining blood-brain interactions in human ICH.

**Electronic supplementary material:**

The online version of this article (10.1186/s12974-019-1433-4) contains supplementary material, which is available to authorized users.

## Introduction

Approximately 795,000 strokes occur in the USA each year [1]. Stroke remains a leading cause of death and disability [2, 3]. Although primary non-traumatic intracerebral hemorrhage (ICH) only accounts for 10–15% of strokes [1, 4], its mortality rate of 59% at 1 year [4] is much higher than that for ischemic stroke (IS) (14%) [5].

Many pre-clinical and a few clinical studies implicate neuroinflammation as contributing to neurological injury produced by ICH [3, 6, 7]. Following ICH, a complex cascade of local and systemic immune responses occurs leading to blood-brain barrier disruption, cerebral edema, and cell damage/death followed by hematoma removal and brain repair [7–11]. Since there is communication between the peripheral immune system and the central nervous system through afferent and efferent trafficking of cells and molecules, the peripheral immune system is a key driver of damage and repair following ICH [7, 12, 13]. Peripheral leukocytic infiltration into the brain is seen early following ICH [7]. This involves chemokines, cytokines, matrix metalloproteinases (MMPs), and interactions between circulating leukocytes and vascular endothelial cells, with different cell types employing common as well as unique pathways [14]. Thus, it is important to study both the local and systemic immune response to human ICH.

We examined the systemic peripheral immune response to human ICH and how it compares to the local human perihematomal brain tissue response. We investigated the peripheral blood gene co-expression networks and their most interconnected genes (hubs) following ICH, identified differential gene-level RNA expression and the potentially affected pathways, and compared the peripheral blood response to the one in previously published human peri-hematoma ICH brain [15]. The hub genes we identified have been implicated as major regulators of the immune response including the inflammasome, autophagy, and transcriptional and epigenetic regulation. Some of the hub genes have also been successfully tested in animal ICH models. Notably, there was a significant overlap between genes differentially expressed in this human ICH peripheral blood study compared to a prior human study of perihematomal brain tissue [15]. These findings will further our understanding of the biological processes occurring following ICH, as well as provide human data confirming targets that have been tested or will be tested in animal ICH models. The findings also highlight candidate genes that influence major processes underlying the response to ICH and parallel responding genes/pathways in blood and brain, underscoring the importance of further studies of the peripheral blood-brain immune communication.

## Materials and methods

### Study subjects

Sixty-six male (M) and female (F) subjects with intracerebral hemorrhage (ICH, *n* = 33, 24 M/9F) and vascular risk factor (VRF)-matched control subjects (CTRL, *n* = 33, 24 M/9F) were recruited from the Universities of California at Davis and San Francisco as well as the University of Alberta, Canada. Procedures were approved by the IRBs at participating universities and written informed consent was obtained from participants or their proxy. ICH was diagnosed by board-certified neurologists based upon histories, exams, and magnetic resonance imaging (MRI) and/or computed tomographic (CT) brain scan [16]. Subjects were matched for age, race, sex, and VRFs, which included hypertension, diabetes mellitus, hyperlipidemia, and smoking status. Exclusion criteria were previous stroke (for CTRLs) and ischemic stroke with hemorrhagic transformation.

### Blood collection, RNA isolation

Blood was collected in PAXgene tubes and RNA isolated as previously described [17, 18]. There was a single blood draw per subject. Time after symptom onset for ICH varied from 4.2 to 124.3 h.

### Arrays and processing

RNA was processed on the GeneChip® Human Transcriptome Array (HTA) 2.0 (Affymetrix, Santa Clara, CA) which examines the coding and non-coding transcriptome. Raw expression values for each gene were saved in Affymetrix.CEL and Affymetrix.DAT files. Using Affymetrix Power Tools (APT), the. CEL transformer applied GC correction (GCCN) and single space transformation (SST) to the HTA.CEL files. GCCN-SST transformations were conducted through a command line using APT 1.18.0 in “batch” mode. GCCN-SST transformed .CEL files were uploaded into Partek Flow software (Partek Inc., St. Louis, MO) and probe sets mapped to the Human Genome hg19 using the STAR 2.4.1d aligner. Nominal read coverage depth was defined as 30 million and default mapping parameters were used. RPKM normalization was performed.

### Weighted gene co-expression network construction and analysis

The 56,638 genes (mRNA and ncRNA) present in ICH and CTRL data were filtered by removing genes with a maximum expression across all samples of < 5, leaving 29,513 genes. Then, genes with expression < 3 in greater than 50% of the male and female samples across all groups were removed, leaving 21,175 genes. This was done to remove potential outliers, reduce noise, decrease sex bias due to an uneven number of males and females in the data, and focus analyses on the most robust correlations. Data were then imported into R and the function *goodSamplesGenes* within the WGCNA package used to identify any missing values or zero-variance genes to be removed from the sample. The CTRL and ICH data were then processed in R using WGCNA [19].

WGCNA identified Pearson correlations throughout the data to develop modules of co-expressed genes. An approximate scale-free topology was depicted by the data, as is expected of gene co-expression networks [20]. To maximize strong correlations between genes, we assigned a soft-thresholding power β = 8 since it was the lowest β with the highest *R*^2^ value that passed the 0.8 threshold (Additional file 1: Figure S1). Due to the large number of genes being processed, we set the minimum module size to 100 genes. Rather than using a static cut-off during module construction, we utilized the *cutreeDynamic* function to form modules because of its ability to identify nested modules within complex dendrograms [21]. Additional parameters within *cutreeDynamic* included method = “tree” and deepSplit = FALSE. To focus on genes of likely greatest importance in ICH, we identified those with the top 5% highest membership to their respective module [22]. These hub genes are highly interconnected within the module and their interconnectivity was quantified by kIN—the gene’s intramodular connectivity [23]. Significant modules with respect to diagnosis (ICH, CTRL) were extracted (*p*(Dx) < 0.05) for additional processing.

### Differential gene expression

Differentially expressed genes were derived using Partek Genomics Suite 7.17 software (Partek Inc., St. Louis, MO) on the complete post-filtered dataset of 21,175 genes. A three-way ANCOVA model with REML (restricted maximum likelihood) and the factors diagnosis, scan date, sex, age, time, and an interaction between diagnosis and sex was used. Genes passing a FDR (false-discovery rate) *p* value < 0.05 and a fold change (FC) > |1.2| for ICH vs CTRL were considered significant.

### Cell-specific gene involvement

To identify modules of co-expressed genes enriched with blood cell type-specific genes, we overlapped the gene list of each module with lists of blood cell type-specific genes [24, 25]. We calculated significant overlaps of genes using hypergeometric probability testing with the R function *phyper.* Those with a *p* < 0.05 were deemed significant.

### Pathway and gene ontology analyses

Hub genes and modules significant for diagnosis were further analyzed with ingenuity pathway analysis (IPA) [26] and the DAVID Bioinformatics Database (DAVID 6.8) [27]. Module genes were input into IPA to map them to molecular functions, associated diseases, and canonical pathways [28] using Fisher’s exact test *p* < 0.05 and a multiple-comparison correction via the Benjamini-Hochberg procedure (adjusted *p* < 0.05). The same lists of module genes were input into DAVID for gene ontology (GO) analysis (EASE adjusted Fisher’s exact test *p* < 0.05).

### Network visualization and hub gene identification

The R function within WGCNA *visantPrepOverall* generated a list of intramodular gene connections that were imported into VisANT to visualize the networks [29, 30]. To better capture hub gene connectivity, parameters modified within the *visantPrepOverall* function were numint = 10,000 and signed = FALSE. After importing the data into VisANT, the minimum weight cutoff was adjusted to display a visually distinguishable number of connections. The lengths and colors of the edges (connections) in the images are arbitrary. Networks were created with VisANT for modules deemed significant with *p*(Dx) < 0.05 and for significant hub gene networks within them.

## Results

### Demographic and clinical characteristics

Subjects’ demographic and clinical characteristics are listed in Additional file 2: Table S1 [18]. There were no significant differences in hypertension, diabetes mellitus, hyperlipidemia, or smoking for ICH compared to CTRL subjects. Time since ICH to blood draw was 57.3 ± 30.6 h (± standard deviation). The mean age of ICH subjects was 62 ± 14.3 years (± standard deviation) and was not significantly different from 63.5 ± 13.1 years for CTRL.

### WGCNA: ICH and CTRL analysis

#### Module assignments

**Fig. 1:**
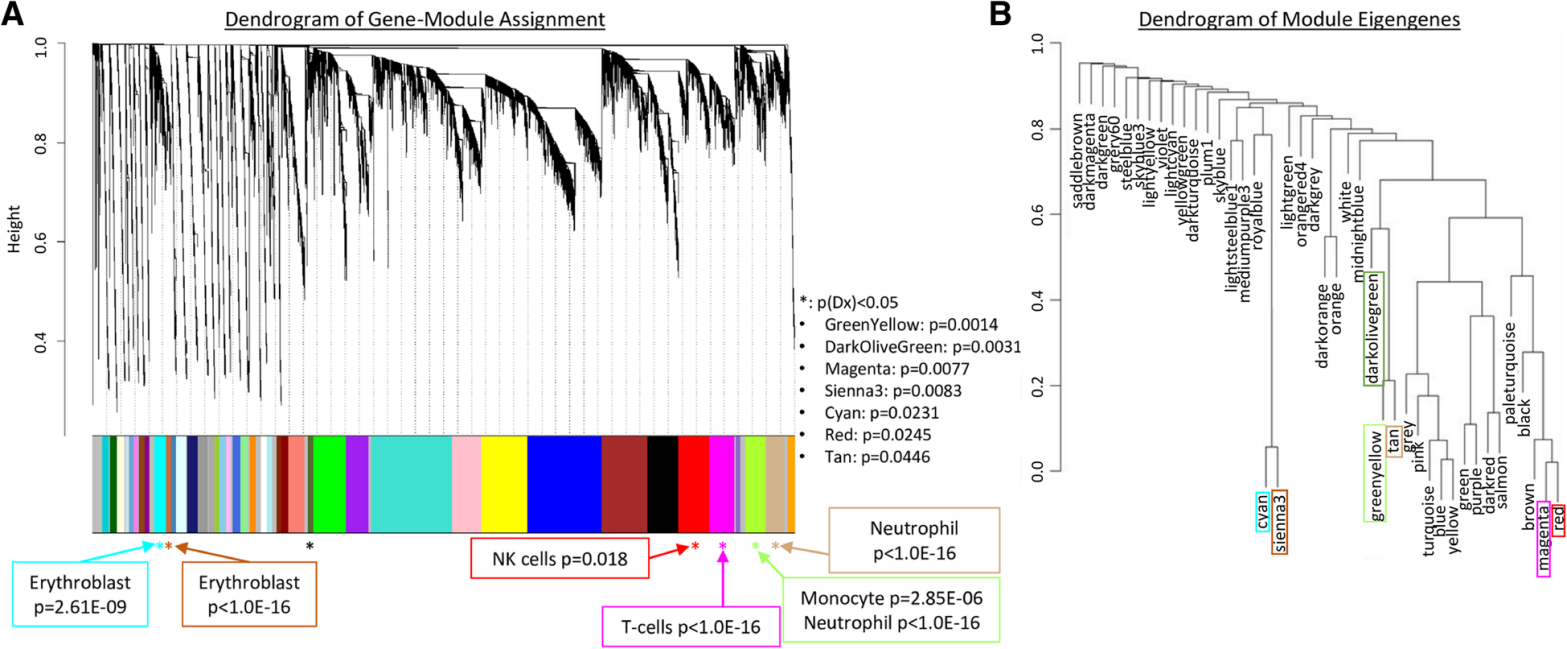
A dendrogram of the genes and their module assignments (**a**); a dendrogram of only the module eigengenes (representative of the overall gene expression within that module) (**b**). The significant modules in respect to Dx (cyan, darkolivegreen, greenyellow, magenta, red, sienna3, and tan) are marked with asterisks in (**a**) and colored in module-corresponding colors in (**b**). Dx-specific modules with significant overlap/enrichment with cell-specific genes are labeled in (**a**) with the specific cell type, and the hypergeometric probability *p* value of overlap between the module’s gene list and the cell type-specific gene list from Watkins et al. [31] and T cell-specific genes from Additional file 2: Table S1 from Chtanova et al. [32] is presented

Analysis of the 66 samples (33 CTRL and 33 ICH) for 21,175 genes identified 41 modules of co-expressed genes. Modules ranged in size from 2416 (turquoise) to 114 genes (lightsteelblue1). A gene dendrogram and their modules are shown in Fig. 1a. A dendrogram of the module eigengenes (representative of the overall gene expression within that module) is shown in Fig. 1b. Genes and their module assignments are listed.

in Additional file 2: Table S2. Of the 41 modules, 7 showed a significant difference between ICH and CTRL groups with *p*(Dx) < 0.05 (cyan, darkolive green, greenyellow, magenta, red, sienna3, and tan) (marked with asterisks in Fig. 1a and colored in module-corresponding colors in Fig. 1b).

#### Cell type involvement

We then compared the genes in the ICH-associated modules (*p*(Dx) < 0.05) to genes reported to be expressed in normal human erythroblasts, megakaryocytes, B cells, cytotoxic and helper T cells, natural killer cells, granulocytes, or monocytes [24, 25]. There was significant overlap/enrichment in six of the seven ICH modules (Fig. 1a). Cyan and sienna3 were enriched for erythroblast-specific genes (*p* = 4.9 × 10^−10^ and *p* < 1 × 10^−16^, respectively); tan—with CD66b^+^/neutrophil-specific genes (*p* < 1 × 10^−16^); and greenyellow—with CD14^+^/monocyte and CD66b^+^/neutrophil-specific genes (*p* = 2.9 × 10^−06^ and *p* < 1 × 10^−16^, respectively). Magenta was enriched with genes selectively expressed in T cells (*p* < 1 × 10^−16^), and red with CD56^+^/natural killer cell-specific genes (*p* = 1.8 × 10^−2^). Lists of these genes and their modules are in Additional file 2: Table S3.

#### Central intramodular hub genes

**Fig. 2:**
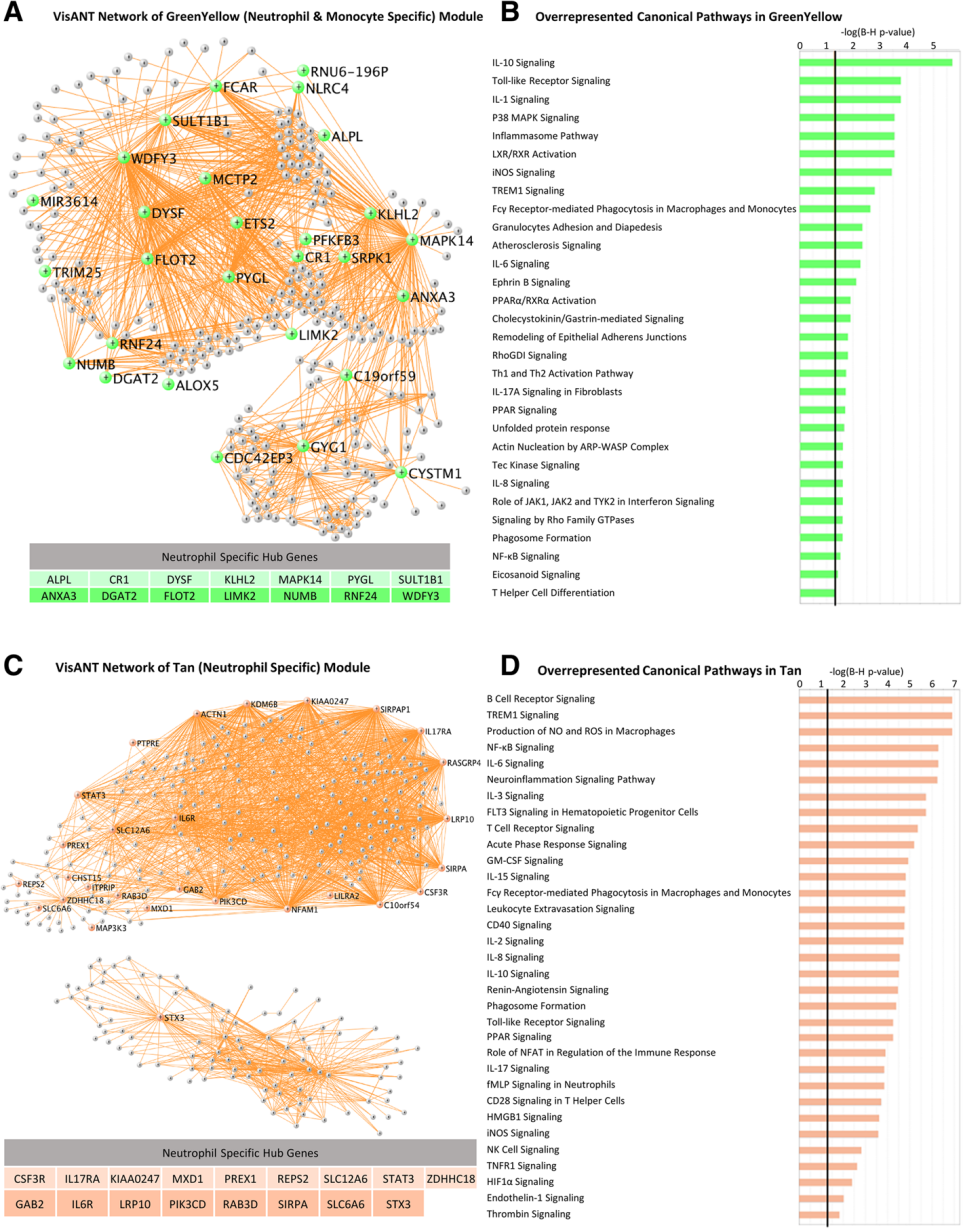
Greenyellow module. **a** VisANT Network of greenyellow (neutrophil- and monocyte- specific) module. The hub genes are colored in green-yellow. Neutrophil-specific hub genes are listed below. **b** Overrepresented canonical pathways in greenyellow (IPA analysis, Benjamini-Hochberg (B-H) corrected *p* < 0.05). *X*-axis: negative Log_10_-transformed p-value for significance for enrichment in specific IPA pathways. Pathways higher than the black vertical line (-Log_10_(B-H *p*))  > 1.3 (which corresponds to *p*(B-H *p*) < 0.05) are significant. **c**, **d** Tan module. Hub genes are colored in tan

**Fig. 3:**
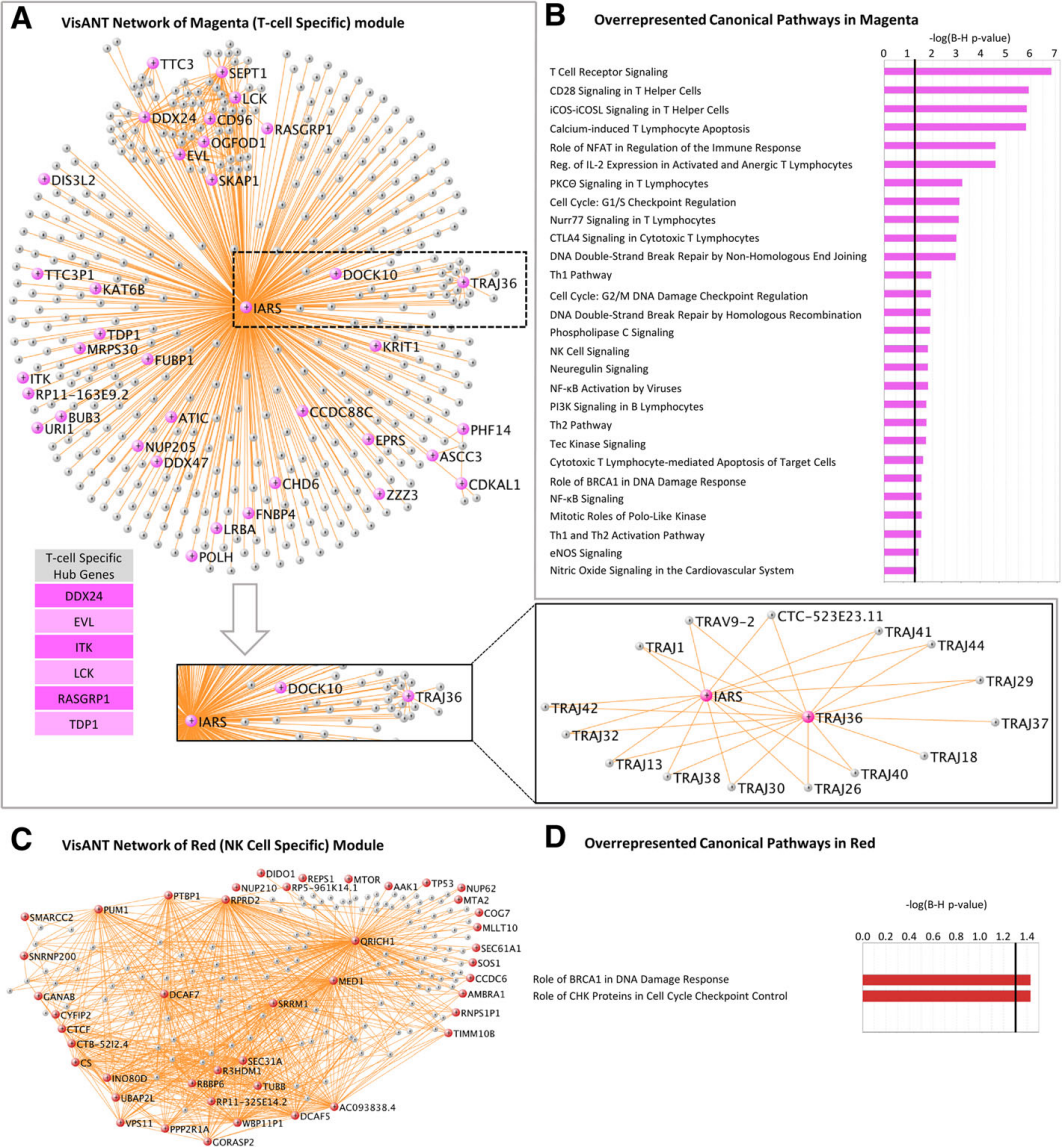
**a**, **b** VisANT network of magenta (T cell specific) module. The Hub genes are colored in magenta. T cell specific hub genes are listed below. **b** Overrepresented canonical pathways in magenta (IPA analysis, B-H corrected *p* < 0.05). *X*-axis: negative Log_10_-transformed *p* value for significance for enrichment in specific IPA pathways. Pathways higher than the black vertical line (-Log_10_(B-H *p*))  > 1.3 (which corresponds to *p*(B-H *p*) < 0.05) are significant. **c**, **d** Red module. Hub genes are colored in red

**Fig. 4:**
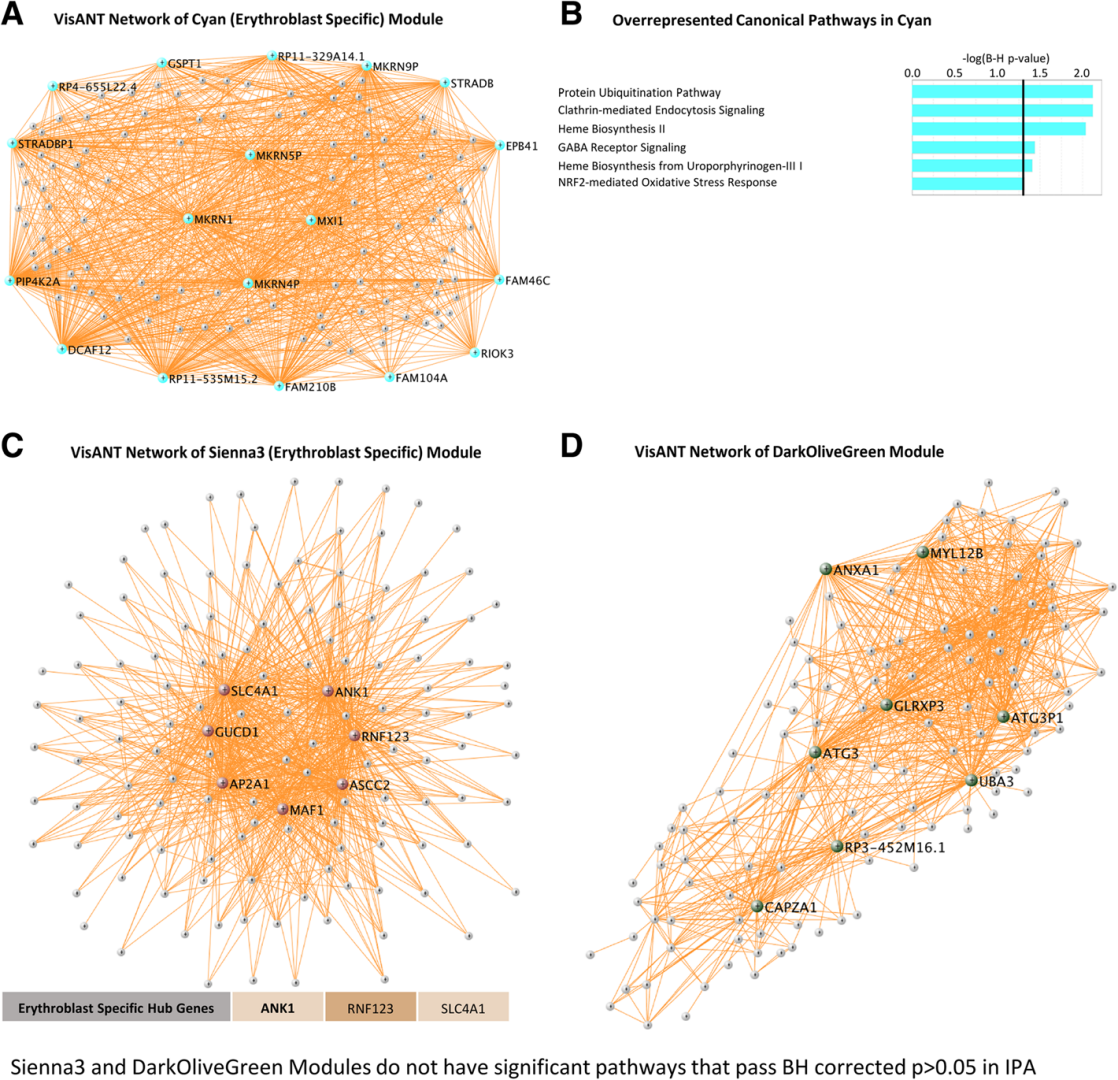
**a**, **b** VisANT network of cyan (erythroblast-specific) module. The hub genes are colored in cyan. **b** Overrepresented canonical pathways in cyan (IPA analysis, B-H corrected *p* < 0.05). *X*-axis: negative Log_10_-transformed *p* value for significance for enrichment in specific IPA pathways. Pathways higher than the black vertical line (-Log_10_(B-H *p*))  > 1.3 (which corresponds to *p*(B-H *p*) < 0.05) are significant. **c** VisANT network of sienna3 module. Hub genes are colored in sienna3. No IPA pathways passed B-H corrected *p* < 0.05. **d** VisANT network of darkolivegreen module. Hub genes were colored in dark olive green. No IPA pathways passed B-H corrected *p* < 0.05

Hub genes may be important targets for treating ICH. A list of hub genes for each module is in Additional file 2: Table S4 and the canonical pathways for those hub genes are listed in Additional file 2: Table S5. The VisANT networks of the ICH-associated modules, including their hub genes and the biological processes that are overrepresented in each module are depicted in Figs. 2, 3, and 4. Figures 2a, c, 3a, c, and 4a, c, d show the networks of the seven ICH diagnosis-significant modules, where the enlarged, labeled, and colored (non-gray) nodes are the module’s hub genes. Not all genes and connections are displayed since inclusion of too many generated an incomprehensible image.

**Fig. 5:**
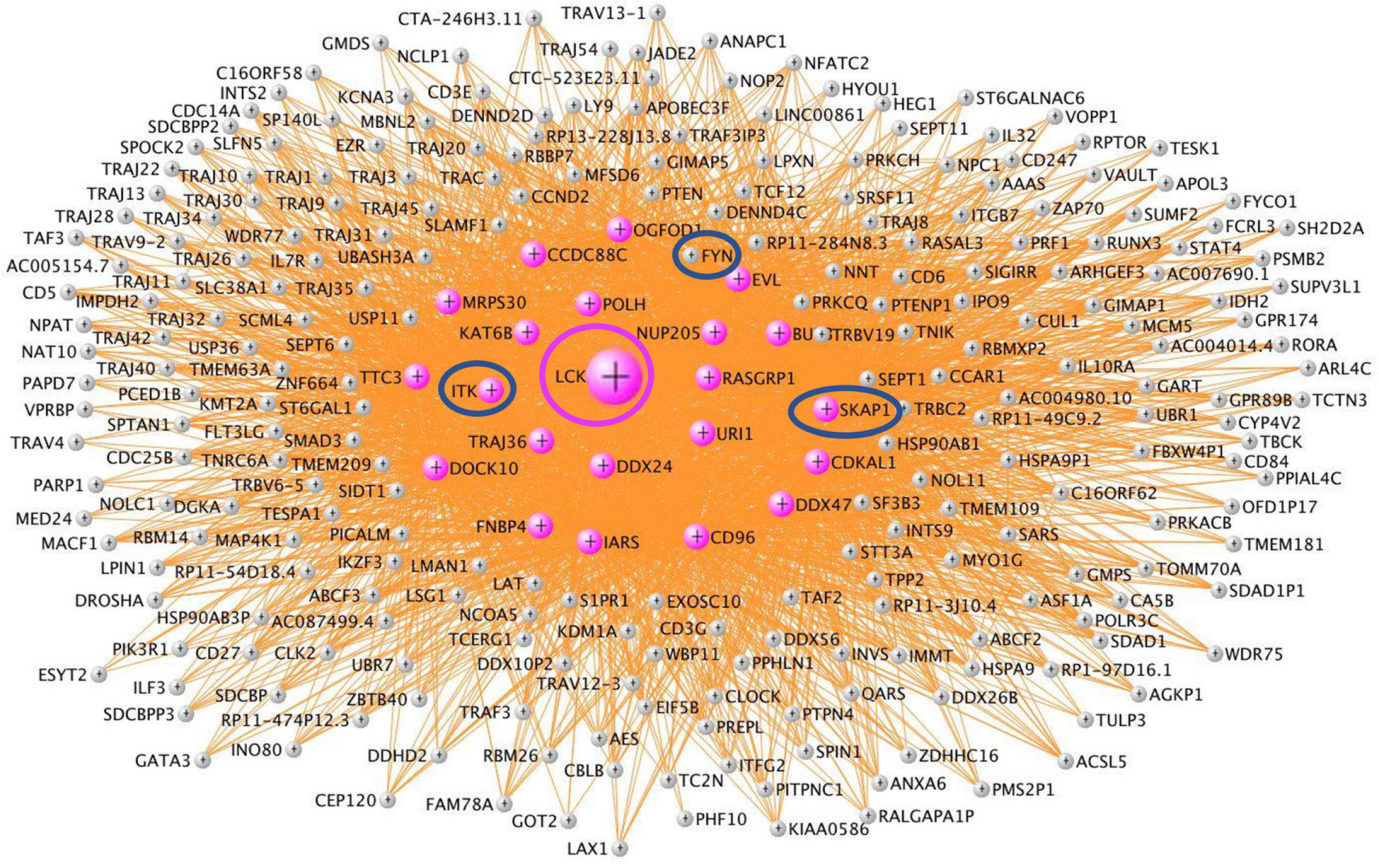
LCK network in the magenta module. Genes colored in magenta are hub genes. Note other Src kinases (like FYN and ITK) and Src kinase-associated protein (SKAP1) are circled

**Fig. 6:**
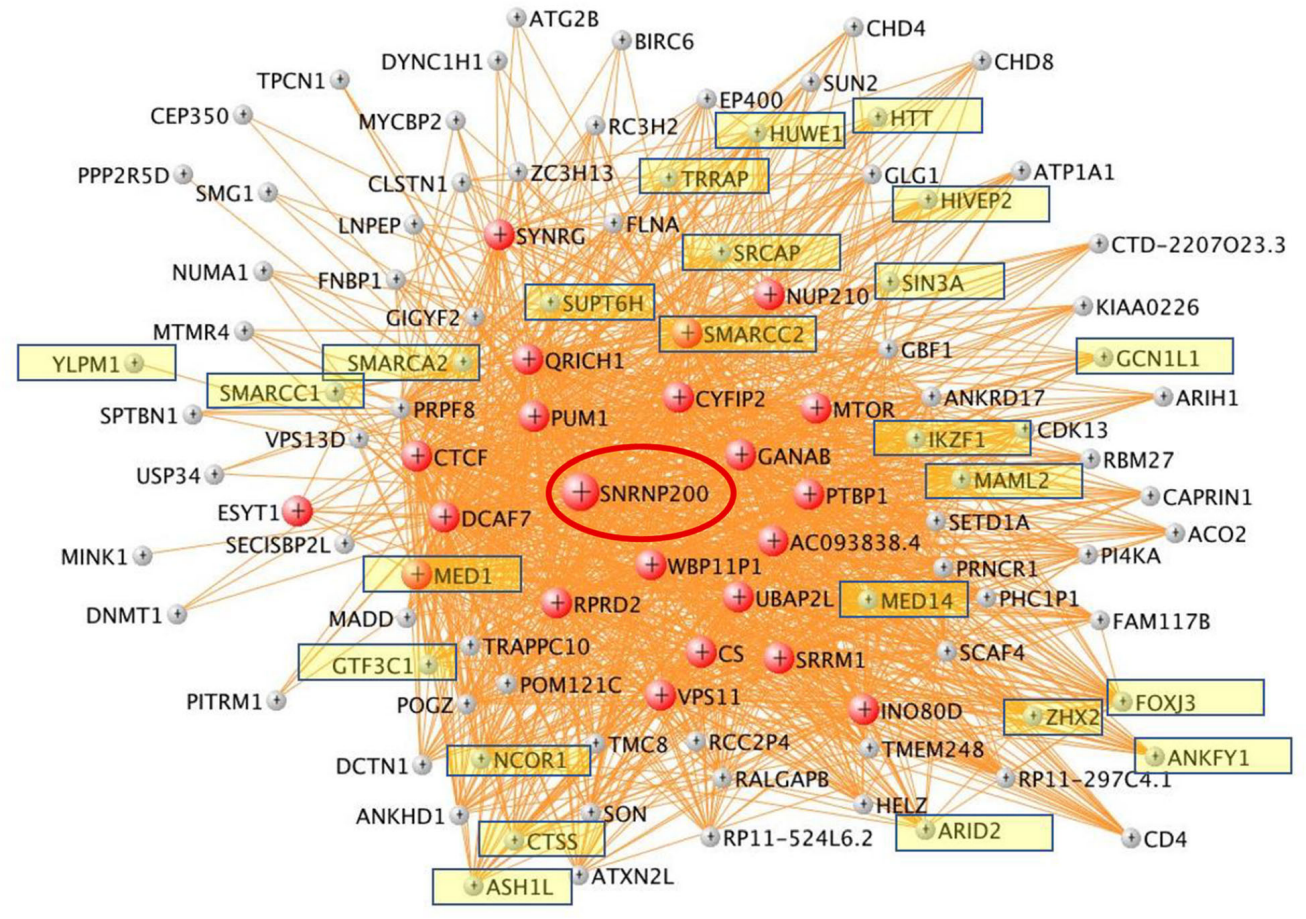
SNRNP200 network in the red module. Genes colored in red are hub genes. Yellow highlighted genes are transcription regulators

**Fig. 7:**
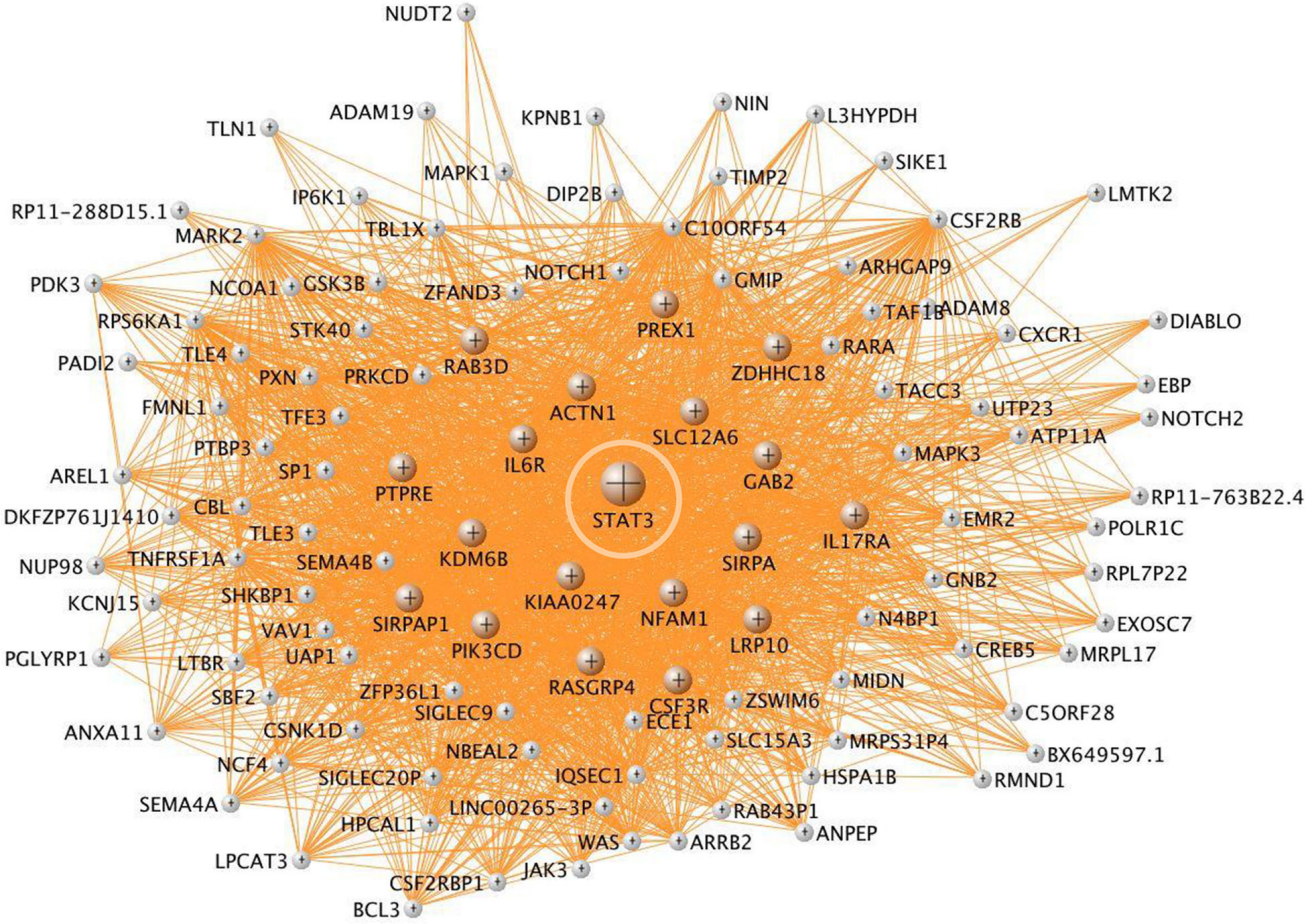
STAT3 network in the tan module. Genes colored in tan are hub genes

The most statistically significant hub genes included isoleucyl-TRNA synthetase (IARS) (Fig. 3a; Additional file 1: Figure S2); sphingosine-1 phosphate receptor 1 (S1PR1) (Additional file 1: Figure S3); T cell receptor alpha joining 36 (TRAJ36) (Fig. 3a); proto-oncogene, Src family tyrosine kinase (LCK) (Fig. 5); IL2 inducible T cell kinase (ITK) (Additional file 1: Figure S4); mediator complex subunit 1 (MED1), mediator complex subunit 14 (MED14), small nuclear ribonucleoprotein U5 subunit 200 (SNRNP200) (Fig. 6), mechanistic target of rapamycin kinase (mTOR), autophagy nd beclin 1 regulator 1 (AMBRA1) (mTOR and AMBRA1 networks) (Additional file 1: Figures S5 and S6); NLR family CARD domain containing 4 (NLRC4), WD repeat and FYVE domain containing 3 (WDFY3), microRNA 3614 (miR3614) (Additional file 1: Figures S7, S8, and S9); autophagy related 3 (ATG3) (Additional file 1: Figure S10); solute carrier family 4 member 1 (Diego blood group) (SLC4A1), ankyrin 1 (ANK1) (Additional file 1: Figures S11 and S12); phosphatidylinositol-5-phosphate 4-kinase type 2 alpha (PIP4K2A) (Additional file 1: Figure S13); colony stimulating factor 3 receptor (CSF3R) (Additional file 1: Figure S14); and signal transducer and activator of transcription 3 (STAT3) (Fig. 7). The *p* values for diagnosis for the hub genes in all modules are in Additional file 2: Table S6a.

There were two non-coding RNA hub genes: SNRNP200 (Fig. 6) and microRNA miR3614 (Additional file 1: Figure S9). Of the modules with a significant association to ICH diagnosis, DarkOliveGreen (the only module not associated with a specific cell type) was the only module that also showed an association to the time since stroke onset. The *p* values for time-since-event for all modules are shown in Additional file 2: Table S6b.

The hub genes for each module differed by cell type and function. Notably, 14 out of 31 of the hub genes in the greenyellow module were neutrophil-specific (Fig. 2a). More than half of the hub genes in the tan module were neutrophil-specific (17 out of the 28) (Fig. 2c). Six hub genes in magenta (Fig. 3a) were T cell specific. Three hub genes in sienna3 (erythroblast module) were erythroblast-specific (Fig. 4c). Table 1 shows all hub genes in the ICH-significant modules; Table 2 shows three representative hub genes for each module and their relevant functions.

**Table 1:**
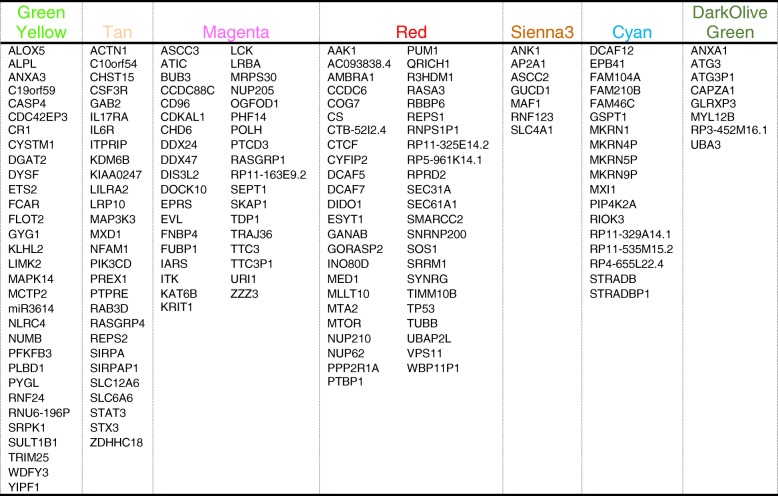
Hub genes in the ICH-significant modules

**Table 2:**
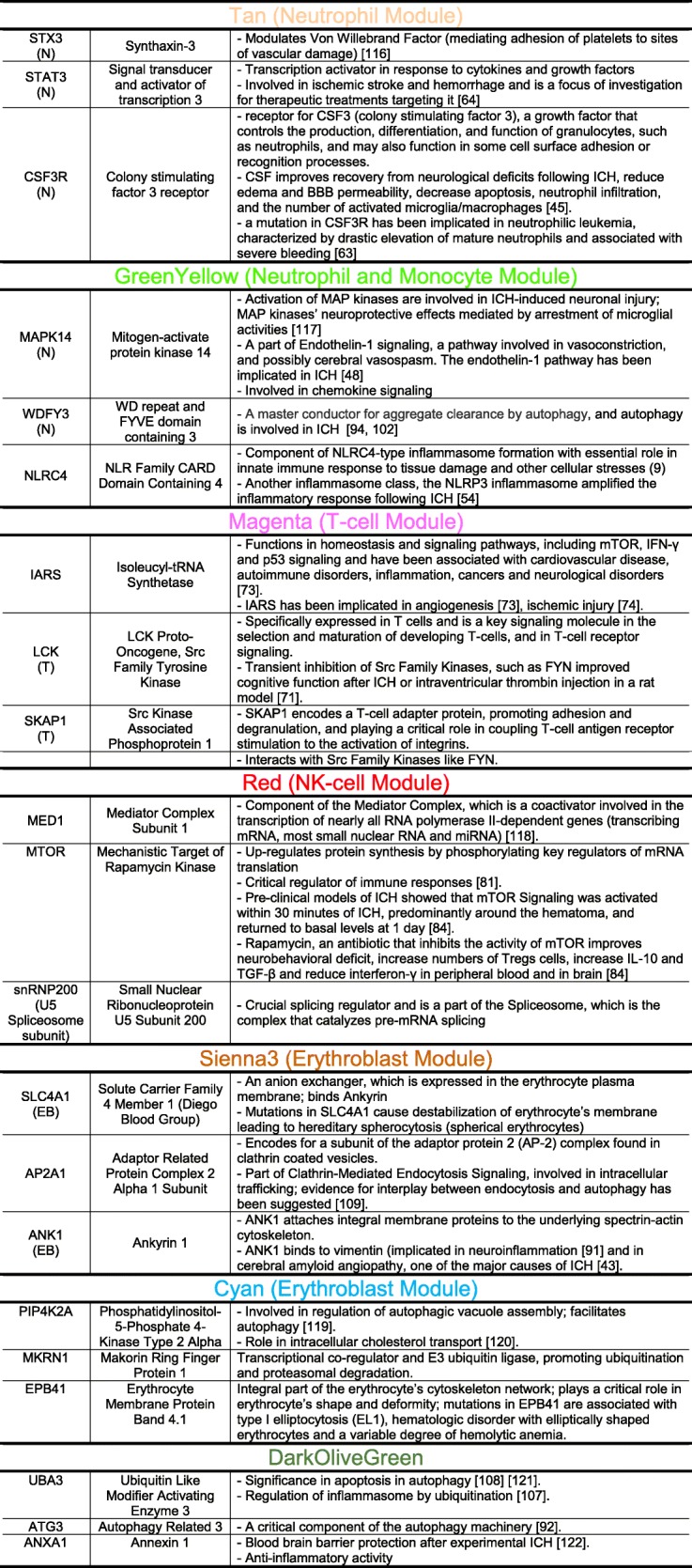
Selected three relevant hub genes and their relevant functions from each module. N—neutrophil-specific gene, M—monocyte-specific gene, T—T cell-specific gene, EB—erythroblast-specific genes [43, 45, 48, 54, 63, 64, 71, 73, 74, 81, 84, 91, 92, 94, 102, 107–109, 116–122]

#### Pathway analyses

A list of pathways associated with each module is in Additional file 2: Table S7. Selected pathways in the ICH-associated modules are shown in Figs. 2b, d, 3b, d, and 4b. The greenyellow module (neutrophil/monocyte module) included toll-like receptor (TLR), inflammasome, and PPAR signaling which are all involved in neuroinflammation (Fig. 2b). The tan module (neutrophil module) included leukocyte extravasation signaling (Additional file 1: Figure S15), several interleukin-signaling pathways, T and B cell receptor signaling, apoptosis pathways, NF-κB signaling, and PPAR signaling. Tan module functions included renin-angiotensin and endothelin-1-signaling related to vasoconstriction/vasodilation; and coagulation pathways such as thrombin signaling (Fig. 2d).

The magenta module was mainly associated with T cell receptor signaling (Additional file 1: Figure S16) and pathways involving T lymphocytes (Fig. 3b) (CD28 signaling in T helper cells, iCOS-iCOSL signaling in T helper cells, Th1 and Th2 pathways, calcium-induced T lymphocyte apoptosis, PKCθ, and Nur77 signaling in T lymphocytes). The red module (natural killer cell module) was overrepresented with genes involved in DNA repair and cell cycle regulation (Fig. 3d). The cyan module (erythroblast module) was associated with the NRF2-mediated oxidative stress response, protein ubiquitination, and clathrin-mediated endocytosis, which are all pathways implicated in hematoma clearance following ICH [31–33], as well as heme biosynthesis pathways (Fig. 4b).

**Fig. 8:**
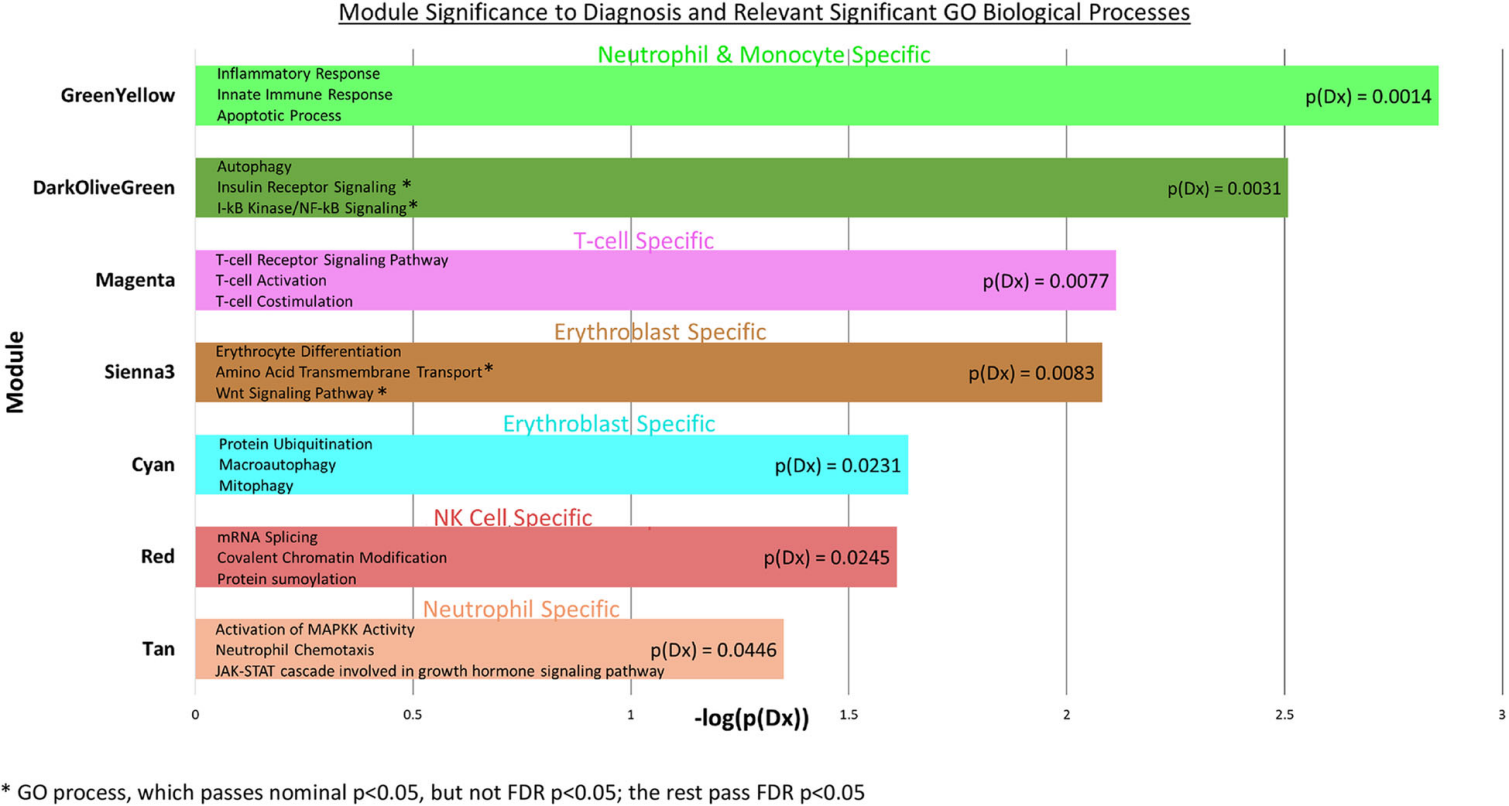
Module significant to diagnosis (Dx) and relevant gene ontology (GO) biological processes. Top 3 relevant biological processes presented in each bar. *X*-axis: negative Log_10_-transformed *p* value for significance with Dx for each significant module. Linear *p*(Dx)-value displayed in each bar. Significant over-representation for cell-type specific genes displayed above each module. *GO process, which passes nominal *p* < 0.05, but not FDR *p* < 0.05; the rest pass FDR *p* < 0.05

The statistically significant processes (*p* < 0.05) using gene ontology (DAVID functional annotation tool) are shown in Additional file 2: Table S8. The top 3 in each module are listed in Fig. 8. Some of the processes not mentioned above included apoptosis and autophagy (greenyellow), insulin receptor signaling (darkolivegreen), amino acid transport and Wnt signaling (sienna3), protein ubiquitination and macroautophagy and mitophagy (cyan), mRNA splicing and protein sumoylation (red), and MAPKK and JAK-STAT signaling (tan).

#### Gene-level expression in blood compared to perihematomal brain

Of the 21,175 genes examined in this study, 1225 showed differential gene-level expression between ICH and CTRL (FDR *p* < 0.05, FC > |1.2|; Additional file 2: Table S9a). There were 63 hub genes (Table 1) from the 7 modules associated with ICH (Additional file 2: Table S9b). The most significant pathways associated with the 1225 genes were T and B cell receptor signaling, Th1 and Th2 activation, neuroinflammation, and IL-1, -2, -6, -8, -10, and -12 signaling (Additional file 2: Table S9c). The 1225 gene list was enriched with genes associated with neutrophils (*p* < 1 × 10^−16^) and T cells (*p* = 1.9 × 10^−3^) (Additional file 2: Table S9d). There was neutrophil enrichment in greenyellow and tan modules (*p* = 5.6 × 10^−13^ and *p* = 4.6 × 10^−4^, respectively), and T cell enrichment in magenta (*p* = 3.1 × 10^−06^) (Additional file 2: Table S9e).

**Fig. 9:**
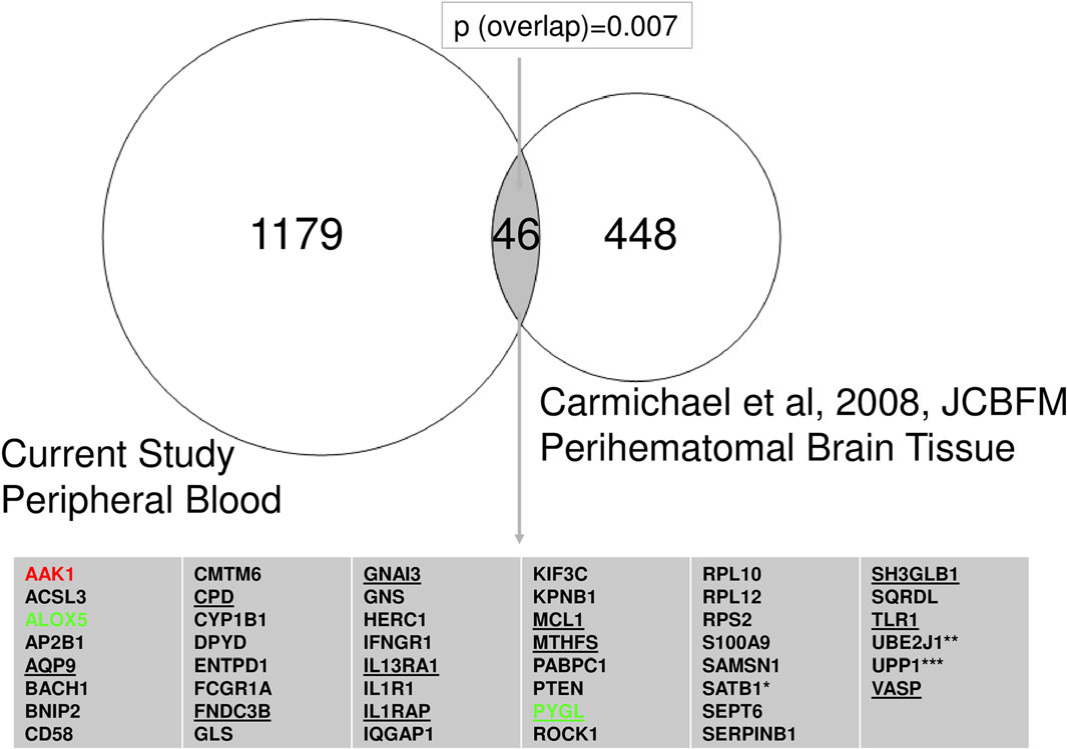
Overlap between differentially expressed genes in peripheral blood (current study) and perihematomal brain tissue [15] between patients with ICH and controls. There was significant enrichment (*p* = 6.4 × 10^−08^) of neutrophil-specific genes in the overlap of 46 genes. The underlined genes are neutrophil-specific (based on the HeamAtlas [24]). * SATB1 (SATB Homeobox 1)—T cell-specific based on [25]. ** UBE2J1 (ubiquitin conjugating enzyme E2 J1)—B lymphocyte-specific based on [24]. *** UPP1 (uridine phosphorylase 1)—Monocyte-specific based on [24]. The green yellow genes ALOX5 (arachidonate 5-lipoxygenase) and PYGL (glycogen phosphorylase L) are hub genes in the greenyellow ICH module. The red gene AAK1 (AP2 Associated Kinase) is a hub gene in the red ICH module

By assessing gene-level expression in blood of ICH subjects, we were able to compare our study to another that examined human perihematomal brain tissue [15]. Forty-six of our blood-derived differentially expressed genes (DEGs) overlapped with the list of 534 DEGs from the brain ICH study (*p* = 0.007; Additional file 2: Table S9f) (Fig. 9). The 46 DEGs showed enrichment for neutrophil-specific genes (6.4 × 10^−08^; Additional file 2: Table S9g). The 46 genes were involved in interleukin (1, 6, 8, and 10) signaling, neuroinflammation, apoptosis, and PPAR signaling (Fisher’s Exact *p* < 0.05; Additional file 2: Table S9h).

## Discussion

Seven modules of co-expressed genes were associated with ICH. Six were enriched in genes predominantly expressed in specific cell types suggesting cell-specific coordination of the peripheral immune response to ICH. This confirms many experimental studies of a complex immune response to ICH [7, 34]. The identified hub genes are biologically relevant, involved in major pathways including immune, autophagy and transcriptional, post-transcriptional, and epigenetic regulation and hematoma clearance. A number of these pathways have also been reported in experimental ICH studies [7, 34]. The data highlights the utility of systems-level approaches to studying human ICH.

### Neutrophil and monocyte modules (greenyellow and tan modules)

Neutrophils are among the first peripheral blood cell type to infiltrate perihematomal brain and vessels and the hematoma itself following ICH [35]. Neutrophils contribute to blood-brain barrier (BBB) breakdown, chemoattraction, activation of resident microglia, and brain injury at early times following ICH [36]. Later, IL-27 shifts neutrophils from producing pro-inflammatory/cytotoxic products to production of beneficial iron-scavenging molecules like lactoferrin [37]. IL-27 and lactoferrin reduced edema, enhanced hematoma clearance, and improved neurological outcomes in experimental ICH [37]. Peripheral monocytes also enter the brain after ICH and eventually outnumber neutrophils [7]. They also appear to have adverse effects early, but then contribute to tissue repair and hematoma phagocytosis later after ICH [7].

Our data revealed two modules enriched in neutrophil-specific genes and one of these was also enriched in monocyte-specific genes. The greenyellow (neutrophil and monocyte) module included IL-6, IL-8, and IL-10 signaling; inflammasome pathway and phagosome formation; toll-like receptor (TLR) signaling; NF-kB and PPAR signaling; and processes involved in leukocyte extravasation through the BBB. Most of these have been implicated in ICH [7]. For example, TLR signaling affects ICH-induced brain injury and outcome [38]. Serum levels of IL-6 and IL-10 increase following ICH [39]. In addition, peroxisome proliferator-activated receptors (PPARs), nuclear hormone receptors that regulate immune/inflammatory responses, have been implicated in ICH and PPARγ agonists improve experimental ICH outcomes [40]. We previously documented differential alternative splicing of PPARγ signaling molecules in ICH [18], which further supports their role in ICH.

Infiltrating neutrophils and monocytes release cytokines, reactive oxygen species (ROS), and matrix metalloproteinases (MMPs), which affect ICH outcomes [41, 42]. Indeed, the greenyellow module included S100A8 (calcium and zinc-binding protein that regulates the immune response); CCR2 (receptor for monocyte chemoattractant protein-1 which mediates monocyte chemotaxis with CCR2 + inflammatory monocytes regulating hematoma clearance and functional recovery following ICH) [7]; PLA2G4A (PLA2G4A deficiency causes platelet dysfunction/bleeding); CD36 (implicated in cerebral amyloid angiopathy, one of the major causes of ICH) [43, 44]; F5 (coagulation factor V, a mutation in which causes Factor V Leiden thrombophilia); and MMP9 (matrix metalloproteinase 9 which causes BBB dysfunction resulting in increased capillary permeability and brain edema following ICH [7, 45]).

Of the 14 neutrophil-specific hub genes in the greenyellow module, those with the highest connectivity included MAPK14, WDFY3 (Additional file 1: Figure S8), SULT1B1, DYSF, FLOT2, ANXA3, PYGL, and KLHL2. Other greenyellow hubs were associated with endothelin-1 signaling (PLBD1, MAPK14, CASP4), which is implicated in cerebral vasospasm following subarachnoid hemorrhage [46]. Serum endothelin-1 levels are elevated in ischemic stroke [47] and ICH [48], and endothelin-1 serum levels correlate with ICH hematoma volumes [48].

Greenyellow hubs were also involved in chemokine signaling (MAPK14, LIMK2), and eicosanoid signaling (PLBD1, ALOX5), which promote inflammation and vascular permeability [49, 50]. The NLRC4 hub (Additional file 1: Figure S7) forms the NLRC4 inflammasome [51, 52]. Inflammasomes, the innate immune system complexes that regulate activation of caspase-1 and IL-1 to induce inflammation in response to infection and host damage molecules (DAMPs), have been implicated in a variety of inflammatory processes [53] including ICH [54]. In a mouse ICH model, another inflammasome class, the NLRP3 inflammasome, amplified the inflammatory response following ICH, suggesting NLRP3 inflammasome inhibition may reduce the ICH inflammatory response [54, 55]. Our data suggest that NLRC4 type inflammasome response may be important in human ICH, and the NLRC4 hub gene could be a therapeutic target.

There was one microRNA (miRNA) hub in the greenyellow module, miR3614 (Additional file 1: Figure S9). Though little is known about this miRNA, our data show that the miR3614 hub is inter-connected with 40 genes (Additional file 1: Figure S9) enriched in IL-6 and IL–10 signaling (MAPK14, IL1R2), granulocyte adhesion and diapedesis (IL1R2, CXCL16), and phagosome formation (FCAR, CR1) (*p* < 0.05). The data suggest an important inflammatory/immune role for miR3614 in human ICH.

The tan module included immune/inflammatory pathways such as leukocyte extravasation signaling (Additional file 1: Figure S15), production of nitric oxide (NO) and ROS, are all implicated in ICH [7]. Tan module gene members included toll-like receptors TLR1, TLR2, TLR4, and TLR6. The tan module also had 17 neutrophil-specific hub genes including IL6R, GAP2, STAT3, and STX3.

The tan module was also enriched in Wnt/β-catenin signaling which has been implicated in BBB maintenance and breakdown and cell apoptosis [56–58]. Treatment with an inhibitor of GSK-3β (glycogen synthase kinase 3 beta), a key molecule in the Wnt/β-catenin signaling pathway, attenuated rtPA-induced hemorrhagic transformation after acute ischemic stroke in rats [59]. Our data revealed significant overrepresentation of Wnt/β-catenin signaling pathway genes (11 genes), including GSK-3β (Additional file 1: Figure S17), and support a role for GSK-3β/Wnt/β-catenin signaling in human ICH.

The tan module was also associated with growth factor signaling, including HGF, NGF, Neutrophin/TRK, GM-CSF, IGF, FGF, renin-angiotensin, VEGF, and JAK-STAT. ICH patients with high serum levels of growth factors such as vascular endothelial growth factor (VEGF), granulocyte-colony stimulating factor (G-CSF), and angiopoietin 1 had good functional outcome and reduced lesion volume [45]. TGFBR1 (TGF beta receptor 1), a tan module gene, when mutated in humans causes the Loeys-Dietz syndrome that is associated with cerebral aneurysms and arterial dissections [60]. Moreover, TGF-β1 promotes recovery after experimental ICH [61]. Modulating JAK-STAT signaling also affects outcomes in experimental ICH [62].

Colony stimulating factor 3 receptor (CSF3R) is a neutrophil-specific Tan hub gene (Additional file 1: Figure S14). It encodes the colony stimulating factor 3 receptor that controls the production, differentiation, and function of granulocytes/neutrophils, and functions in cell surface adhesion or recognition processes. Granulocyte-colony stimulating factor has been investigated as a potential therapeutic target in experimental ICH and shown to improve recovery from neurological deficits, reduce edema and BBB permeability, decrease apoptosis, neutrophil infiltration, and the number of activated microglia/macrophages [45]. A mutation in CSF3R has been implicated in neutrophilic leukemia, characterized by increased neutrophils, and severe bleeding [63]. Several other tan hub genes are involved in growth factor signaling pathways such as PIK3CD, ACTN1, and STAT3. Signal transducer and activator of transcription 3 (STAT3) (Fig. 7) is involved in ischemic stroke and intracerebral hemorrhage and has been a treatment target for experimental ICH [64, 65].

### T cell module (magenta module)

T lymphocytes are part of the adaptive immune system, and depending upon whether they express CD4 or CD8 surface markers, modulate the immune response or elicit cytotoxicity. T lymphocytes have been detected in perihematomal brain tissue of ICH patients, and Treg transfer attenuates neurological deficits in experimental ICH [7]. In addition, the circulating CD4^+^/CD8^+^ T lymphocyte ratio has been suggested as a possible predictor of postoperative intracranial pressure and short-term prognosis [66]. Regulatory T cells ameliorate BBB damage after cerebral ischemia [67] as well as hemorrhagic transformation after tPA administration following ischemic stroke in part by inhibiting CCL2 (monocyte chemoattractant protein 1) and MMP9 [68]. Our previous study showed differential alternatively spliced transcript expression in blood in many T cell receptor genes [18].

The magenta module was enriched in T cell-specific genes, including 54 T cell receptor genes (Additional file 1: Figure S16) that were involved in T cell activation and co-stimulation, CD28 signaling in T helper cells, iCOS-iCOSL signaling in T helper cells, CTLA4 signaling in cytotoxic T lymphocytes, cytotoxic T lymphocyte-mediated apoptosis of target cells, cytotoxic T lymphocyte-mediated apoptosis of target cells, Th1 and Th2 pathways. Our previous study showed that differential expression of a set of alternatively spliced T cell genes distinguished ICH from control subjects using a principal components analysis [18]. Of the 69 T cell receptor transcripts we previously showed were differentially expressed between ICH and control [18], 58 were associated with ICH-associated modules in the current study (44 in magenta, 13 in red, and 1 in tan). This underscores that T cell receptor genes are not only potential biomarkers, differentially expressed at the gene level and differentially alternatively spliced at the transcript level, but are also operating within specific networks that could affect ICH outcomes.

Additional magenta module molecules included Src family kinases (SFK). We have shown that pharmacological inhibition of SFKs improved outcomes and improved BBB function following experimental ICH [69, 70]. Transient inhibition of SFK family member FYN improved cognitive function after intraventricular hemorrhage or intraventricular thrombin injection in a rat model [71]. In our human data, FYN was a magenta module member (Fig. 5), and one of the magenta hub genes is a SFK called LCK (LCK proto-oncogene, Src family tyrosine kinase) (Fig. 5) which is expressed in T cells [25]. LCK is involved in the selection and maturation of developing T cells and in T cell receptor signaling. Another magenta hub is SKAP1 (Src kinase associated phosphoprotein 1), which interacts with SFKs. SKAP1 encodes a T cell adapter protein that promotes adhesion and degranulation, which stimulates T cell antigen receptors to activate integrins.

Another magenta T cell-specific hub was ITK (IL2 inducible T cell kinase). It is a member of the Tec family of non-receptor tyrosine kinases, which regulates the development, function, and differentiation of T cells and NKT cells (natural killer T cells). Upon T cell receptor activation, a series of phosphorylation events recruit ITK to the cell membrane where it is phosphorylated by LCK. The gene networks of these three hub genes—ITK (Additional file 1: Figure S4), LCK (Fig. 5), and SKAP1—were enriched for T cell receptor signaling and other T cell pathways.

The magenta hub with the highest intramodular connectivity was IARS (Fig. 3). IARS belongs to the class-I aminoacyl-tRNA synthetase family which catalyzes the attachment of an amino acid to its cognate tRNA as part of the first step of protein synthesis. Besides its enzymatic function, aminoacyl-tRNA synthases also function in homeostasis and signaling pathways, including mTOR, IFN-γ, and p53 signaling, and have been associated with cardiovascular disease, autoimmune disorders, cancers, and neurological disorders [72]. IARS has been implicated in angiogenesis [73], ischemic injury [74], and inflammation, and the data here suggests IARS might be a novel therapeutic target for ICH.

Sphingosine 1-phosphate receptor 1 (S1PR1), a hub in the magenta module, is connected to 14 genes, notably all of them hubs: CD96, CDKAL1, DDX24, DDX47, DOCK10, EVL, IARS, LCK, MRPS30, OGFOD1, RASGRP1, SEPT1, SKAP1, and TTC3 (Additional file 1: Figure S3). Fingolimod is an FDA-approved S1PR1 immunomodulator treatment for relapsing multiple sclerosis [7, 75]. Fingolimod treatment reduced cell infiltration and brain edema while improving neurological function in experimental ICH [36, 76]. In addition, it improved outcomes in one small human ICH trial [77]. Our data suggests that further studies are warranted not only for S1PR1 but also for its sub-network of 14 hub genes (Additional file 1: Figure S3) for their ICH therapeutic potential.

### RNA splicing, post-translational and epigenetic modifications module—red module

The red module was enriched in natural killer (NK) cell-specific genes, cells for which not much is known about their function in ICH. The red module was also enriched for RNA processing, such as regulation of transcription, mRNA splicing via the spliceosome, as well as epigenetic processes such as chromatin remodeling and covalent chromatin modifications. Post-translational modification processes were also represented, including protein SUMOylation which regulates protein structural and functional diversity by covalent attachment of a member of the small ubiquitin-like modifier (SUMO) family of proteins to lysine residues in specific target proteins. Our previous work showed extensive differential alternative splicing in peripheral blood cells following human ICH [78], thus indicating the importance of transcriptional regulation in human ICH, which is likely immune cell-type specific.

Several red hub genes were involved in regulation of translation as well, which included mechanistic target of rapamycin kinase (mTOR) (Additional file 1: Figure S5). mTOR regulates cellular metabolism, growth, survival, and autophagy [79] in part by upregulating protein synthesis by phosphorylating key regulators of mRNA translation and ribosome synthesis [80]. mTOR also regulates immune responses [81]; controlling the adaptive immune response by promoting differentiation, activation, and function of T cells, B cells, and antigen-presenting cells [81]; differentiation and function of regulatory and effector T cells [82]; and development and activation of NK cells [83]. mTOR signaling is activated around the hematoma within 30 min of experimental ICH, and returned to baseline by one day [84]. Rapamycin, an antibiotic that inhibits mTOR, improves behavioral deficits, increases regulatory T cells, increases interleukin-10 and transforming growth factor-β, and reduces interferon-γ both in peripheral blood and brain following experimental ICH [84]. Our data supports a role for mTOR in human ICH.

Another red module hub gene was a non-coding RNA, small nuclear ribonucleoprotein U5 subunit 200 (SNRNP200) (Fig. 6). SNRNP200 regulates splicing as part of the Spliceosome, which is the complex that catalyzes pre-mRNA splicing. It consists of specialized RNA and small nuclear RNA proteins (snRNPs) U1, U2, U4, U5, and U6, together with 80 other proteins. U5 snRNP contains nine specific proteins and SNRNP200 encodes one of the U5 snRNP-specific proteins. The SNRNP200 network itself contained the hub gene mTOR and 24 out of the 106 genes from the SNRNP200 network were transcriptional regulators (Fig. 6, highlighted), including MED1 (hub), MED14, CTCF (hub), MAML2, SMARCA2, SMARCC1, and SMARCC2 (hub; T cell specific). MAML2 is a transcriptional coactivator for NOTCH proteins [85]. Mutations in the Notch signaling pathway cause heart and vessel disease. Mutations in the NOTCH1 receptor are associated with cardiac disease, while mutations in NOTCH3 cause cerebral autosomal dominant arteriopathy with subcortical infarcts and leukoencephalopathy (CADASIL), which is a vascular disorder with middle-aged onset, associated with ischemic stroke and microhemorrhages [85]. Mediator complex subunit 1 (MED1) and mediator complex subunit 14 (MED14) are components of the mediator complex, which is a coactivator that regulates transcription of nearly all RNA polymerase II-dependent genes (transcribing mRNA, most small nuclear RNA and miRNA). The mediator complex functions as a bridge to convey information from gene-specific regulatory proteins to the basal RNA polymerase II transcription machinery. SMARCA2, SMARCC1, and SMARCC2 regulate gene transcription by altering the chromatin structure around the target genes. Our findings underscore the importance of transcriptional regulation, alternative splicing, and epigenetic mechanisms in the pathophysiology of ICH.

### Erythroblast modules—sienna3 and cyan module

The sienna3 and cyan modules were enriched in erythroblast-specific genes. Erythroblasts are nucleated red blood cells (NRBC), which are progenitors of the enucleate erythrocytes in peripheral blood. NRBCs do not typically cross the barrier between the bone marrow and the peripheral circulation. They are found in peripheral blood under conditions that increase erythropoietic pressure such as inflammation, massive hemorrhage, or severe hypoxia and are associated with poor prognosis and higher mortality [86, 87]. Elevated NRBC have been observed in cerebral hemorrhage in severely growth-restricted infants [88] and in intraventricular hemorrhage in preterm neonates [89]. NRBC are also observed in some cases of sudden infant death syndrome [90].

The sienna3 module had 25 erythroblast-specific genes, including genes involved in congenital hemolytic anemia (ANK1 (ankyrin 1), EPB42 (erythrocyte membrane protein band 4.2), HK1 (hexokinase 1), SLC4A1 (solute carrier family 4 member 1 (Diego blood group)), and SPTB (spectrin beta, erythrocytic)), hereditary spherocytosis (spherical-shaped erythrocytes) (ANK1, EPB42, SLC4A1, SPTB), anemia (ALAS2 (5′-aminolevulinate synthase 2), ANK1, EPB42, HK1, KLF1 (Kruppel like factor 1), SLC2A1, SLC4A1, SPTB), heme biosynthesis (ALAS2), and heme degradation (BLVRB, biliverdin reductase B). The cyan module had 24 erythroblast-specific genes, including ones involved in an iron homeostasis signaling pathway (ABCB10 (ATP binding cassette subfamily B), FECH (ferrochelatase), HBQ1 (hemoglobin subunit theta 1), TFRC (transferrin receptor), and heme biosynthesis (ALAD (aminolevulinate dehydratase), FECH). The expression of erythroblast-specific genes in peripheral blood suggests erythroblasts are present in blood of patients with ICH.

Of the sienna3 hub genes, SLC4A1 (Additional file 1: Figure S11), ANK1 (Additional file 1: Figure S12), and ring finger protein 123 (RNF123) were erythroblast-specific. SLC4A1 encodes an anion exchanger expressed in the erythrocyte plasma membrane. The N-terminal domain in the cytoplasm is an attachment site for the red cell skeleton by binding ankyrin. SLC4A1 mutations cause destabilization of the erythrocyte’s membrane leading to hereditary spherocytosis. ANK1 attaches integral membrane proteins to the underlying spectrin-actin cytoskeleton. ANK1 binds to the erythrocyte membrane protein band 4.2 (EPB42, present in this module) to vimentin which is implicated in neuroinflammation [91] and in cerebral amyloid angiopathy, which is one of the major causes of ICH [43]. ANK1 also links spectrin beta chains (encoded by SPTB, also in this module) to the cytoplasmic domain of SLC4A1 and other exchange proteins.

### Autophagy following ICH

Autophagy involves lysosome-dependent degradation/recycling of cellular components, ranging from proteins to organelles. In macroautophagy, the components are sequestered in a double-membrane-bound organelle, the autophagosome, to eventually fuse with lysosomes for digestion by lysosomal hydrolases [92, 93]. The autophagosome is formed by recruitment of about 15 autophagy-related (ATG) proteins and associated proteins. Autophagy plays a fundamental role in homeostasis, cell death, and in the immune/inflammatory response and its regulation [94]. Dysregulation of autophagy has been implicated in cardiovascular diseases [95], neurodegenerative diseases [96–99], ischemic stroke [100], and experimental ICH [101], reviewed in [102]. Autophagy activation and mitophagy (selective degradation of mitochondria) promoted by voltage-dependent anion channels has also been associated with neuroprotection against cell death following subarachnoid hemorrhage [103]. Autophagy also occurs following experimental cerebral ischemia [104] and ICH [101, 102]. Iron overload from the hematoma plays a key role in ICH-induced autophagy. Infusion of ferrous iron into rat striatum caused autophagy which was reduced by an iron chelator [101]. Neuronal cell death following TBI correlates with autophagy activation and an autophagic clearance impairment [102].

Autophagy-related biological processes populated several of our human ICH modules. The cyan module was significant for macroautophagy, autophagosome assembly, mitophagy [93] (Fig. 8, Additional file 2: Table S8), and TORC2 signaling which regulates autophagy [105]. Major autophagy genes in this module included WIPI2, a component of the autophagosomal machinery that participates in the recruitment of certain ATG (autophagy-associated) proteins; ATG9A, which organizes the pre-autophagosomal structure/phagophore assembly site; and PTEN-induced putative kinase 1 (PINK1), which is involved in selective autophagy with rare mutations being associated with Parkinson’s disease [94].

Autophagy, including mitophagy, plays an important role during erythropoiesis, allowing the proper maturation of red blood cells [106]. The dysfunction of autophagy interferes with the correct erythroid maturation, leading to hematological abnormalities, such as the release of immature red blood cells (erythroblasts) from the bone marrow [106]. In fact, as mentioned above, the presence of circulating erythroblasts in adults reflects increases in erythropoietic activity or failure of the blood filtration mechanism during hematopoietic stress such as inflammation, severe bleeding, hematological malignancy, or severe hypoxia [86].

The cyan module was also enriched in protein ubiquitination pathways which can regulate neuroinflammation [107] and autophagy [108]. This module was also enriched for genes in the NRF2-mediated oxidative stress response pathway, which has been implicated in enhancing the anti-oxidative capacity, phagocytosis, and hematoma clearance following experimental ICH by modulating microglia function following ICH [31]. Clathrin-mediated endocytosis signaling was also enriched, which is involved in intracellular trafficking and may link endocytosis to autophagy [109]. Thus, one might consider the cyan module to be related to hematoma clearance, since it is associated with autophagy, endocytosis, protein ubiquitination, and oxidative stress-response pathways.

Another module enriched in autophagy genes was the darkolivegreen module, where regulation of macroautophagy was the most significant biological process. Moreover, the ATG3 (autophagy related 3) (Additional file 1: Figure S10) hub gene in this module is a component of the autophagy machinery. The darkolivegreen module was also enriched for NF-kB signaling that regulates inflammation, notable since there is a bidirectional regulation between inflammation/NF-kB pathway and autophagy [110]. Indeed, autophagy may regulate ICH-induced neuronal damage via NF-kB [111].

Other hub genes in our data that are key players in autophagy or its regulation include WD repeat and FYVE domain containing 3 (WDFY3, Additional file 1: Figure S8), mechanistic target of rapamycin kinase (mTOR, Additional file 1: Figure S5), VPS11 (VPS11, CORVET/HOPS core subunit), and autophagy and beclin 1 regulator 1 (AMBRA1, Additional file 1: Figure S6). Of these, only mTOR has been shown to play a role in ICH [112]. It is notable that AMBRA1 is required for adult neurogenesis [113], which is impaired after experimental ICH [114].

Since autophagy can be a pro-survival or pro-death mechanism, and may be beneficial or detrimental, more studies are needed to elucidate its role(s) in human ICH. Our results point to a role of autophagy in the peripheral blood cells and their response to human ICH.

### Other blood cell types

Though most modules were enriched in cell type-specific genes, pathways associated with other cell types were also represented in these modules. For example, the most overrepresented pathway in the neutrophil gene-enriched module was B cell receptor signaling. Though a minor role of B cells in ICH-induced brain injury has been suggested [7], our data suggest B cells are involved in the pathophysiology of human ICH. B cells, in addition to producing antibodies, modulate the functions of other immune cells which might be important in ICH [115].

### Blood and brain overlap following human ICH

There was a significant 46-gene overlap from our human blood study and the genes differentially expressed in human brain following ICH [15]. The 46 overlapping genes showed enrichment for neutrophil-specific genes which may be due to infiltrating neutrophils into perihematomal brain tissue. The 46 genes were involved in interleukin 1, 6, 8, and 10 signaling (GNAI3, IL1R1, IL1RAP, ROCK1, IQGAP1, VASP, MCL1), neuroinflammation signaling (GLS, TLR1, IFNGR1, IL1R1), apoptosis (ROCK1, MCL1), PPAR signaling, and leukocyte extravasation signaling (ROCK1, GNAI3, VASP). In addition, three of these blood-brain shared genes (AAK1, ALOX5, and PYGL, Fig. 9) were hub genes in our data. It is plausible they are master regulators in the peripheral immune response and potentially play important role following ICH in perihematomal brain tissue. Thus, they need to be investigated further both in blood and brain. Treatments targeting the 46 shared genes might be considered a priority since this presumably would target peripheral blood immune cells, blood immune cells in brain, and likely brain immune and non-immune cells.

## Limitations

The results need to be validated in a larger study. In addition, the cell-specific expression of each blood cell type is based on their expression in healthy subjects and their cell-specificity may differ in disease. Investigation of isolated cell types will help delineate the contribution of each cell type to the immune response to ICH. Though a given module may be enriched for genes in a specific cell type, there were always genes from other cell types associated with those modules. The role of any given module or hub gene in ICH is difficult to discern from the current study but can often be inferred from experimental ICH studies or the literature.

## Conclusion

Our data revealed inflammatory, regulatory, and autophagy modules of co-expressed genes associated with ICH that were highly enriched with cell-specific genes. This allowed us to identify discrete neutrophil, monocyte, T cell, natural killer cell, and erythroblast gene modules and hub genes. Many of the modules and hub genes either provide novel ICH therapeutic targets or provide human data supporting animal findings for already existing targets. The data also revealed a significant overlap between blood and brain gene responses, underscoring the importance of blood-brain interactions in human ICH.

## Additional files

Additional file 1: **Figure S1.** Soft-thresholding power (A) and connectivity (B). **Figure S2.** IARS network in the Magenta module. Genes colored in magenta are hub genes. **Figure S3.** S1PR1 network within the Magenta module. Genes colored in magenta are hub genes. Note all genes in the S1PR1’s network are hub genes. **Figure S4.** ITK network within the Magenta module. Genes colored in magenta are hub genes. **Figure S5.** mTOR Network in the Red Module. Genes colored in red are hub genes. **Figure S6.** AMBRA1 Network in the Red Module. Genes colored in red are hub genes. **Figure S7.** NLRC4 Network in the GreenYellow Module. Genes colored in green-yellow are hub genes. **Figure S8.** WDFY3 Network in the GreenYellow Module. Genes colored in green-yellow are hub genes. **Figure S9.** miR3614 Network in the GreenYellow Module. Genes colored in green-yellow are hub genes. **Figure S10.** ATG3 Network in the DarkOliveGreen Module. Genes colored in dark green are hub genes. **Figure S11.** SLC4A1 Network in the Sienna3 Module. Genes colored in brown are hub genes. **Figure S12.** ANK1 Network in the Sienna3 Module. Genes colored in brown are hub genes. **Figure S13.** PIP4K2A Network in the Cyan Module. Genes colored in cyan are hub genes. **Figure S14.** CSF3R Network in the Tan Module. Genes colored in tan are hub genes. **Figure S15.** Leukocyte Extravasation Signaling Pathway. Genes circled in purple are present in the Tan Module. **Figure S16.** T cell Receptor Signaling Pathway. Genes circled in purple are present in the Magenta Module. **Figure S17.** GSK-3β Network in the Tan Module. Genes colored in tan are hub genes. (PDF 4696 kb)

Additional file 2: **Table S1.** Subjects' demographic information and clinical characteristics. **Table S2.** List of all 21,175 genes and their respective modules. **Table S3.** Hypergeometric probability testing for all *p*(Dx) < 0.05 modules to all cell-types studied in this document. **Table S4.** List of the top 5% of the most interconnected genes (based on intramodular connectivity - kIN) within each module. Green-highlighted genes are amongst the top 1%. **Table S5.** IPA canonical pathways for only the hubs within modules with *p*(Dx) < 0.05. Pathways in peach color are significant following Benjamini-Hochberg (B-H) correction. Pathways in tan color - only following Fisher’s test. **Table S6.** *p*-values for each module with respect to diagnosis (A) and time since event (B). **Table S7.** IPA canonical pathways for all genes within modules that showed *p*(Dx) < 0.05. Pathways in peach color are significant following Benjamini-Hochberg correction. Pathways in tan color - only following Fisher’s test. **Table S8.** DAVID Gene Ontology results for all modules that showed p(Dx) < 0.05. Pathways in peach color are significant following Benjamini-Hochberg correction. Pathways in tan color - only following Fisher’s test. **Table S9.** List of differentially expressed genes (DEGs) and their respective fold-changes for genes amongst the 21,175 gene list (A). List of hub genes from the 1225 DEGs identified and their respective modules (B). IPA canonical pathways of only the list of 1225 DEGs (C). Cell-type involvement on all 1225 DEGs from this study (D). Cell-type involvement on only hub genes within the 1225 DEGs from this study (E). List of DEGs from this study that overlapped with the DEGs identified in Carmichael, et al. [15] (F). Cell-type enrichment on 46 DEGs that overlapped between this study and Carmichael’s study [15] (G). IPA canonical pathways of only the 46 DEGs that overlapped between this study and Carmichael’s study [15] (H). (XLSX 756 kb)

## Abbreviations

AAK1AP2: Associated kinase 1
ABCB10: ATP binding cassette subfamily B
ACTN1: Actinin alpha 1
ALAD: Aminolevulinate dehydratase
ALAS2: 5′-Aminolevulinate synthase 2
ALOX5: Arachidonate 5-lipoxygenase
AMBRA1: Autophagy nd beclin 1 regulator 1
ANK1: Ankyrin 1
ANXA3: Annexin A3
APT: Affymetrix power tools
ATG: Autophagy related
ATG3: Autophagy related 3
ATG9A: Autophagy related 9A
BBB: Blood-brain barrier
BLVRB: Biliverdin reductase B
CADASIL: Cerebral autosomal dominant arteriopathy with subcortical infarcts and leukoencephalopathy
CASP4: Caspase 4
CCL2: C-C motif chemokine ligand 2
CCR2: C-C motif chemokine receptor 2
CD28: CD28 molecule
CD36: CD36 molecule
CD96: CD96 molecule
CDKAL1: CDK5 regulatory subunit associated protein 1 Like 1
CR1: Complement C3b/C4b receptor 1 (Knops Blood Group)
CSF: Colony stimulating factor
CSF3R: Colony stimulating factor 3 receptor
CT: Computed tomographic
CTCF: CCCTC-binding factor
CTLA4: Cytotoxic T lymphocyte associated protein 4
CTRL: Control subjects
CXCL16: C-X-C motif chemokine ligand 16
DAMP: Damage molecules
DDX24: DEAD-Box Helicase 24
DDX47: DEAD-Box Helicase 47
DEG: Differentially expressed genes
DGAT2: Diacylglycerol O-acyltransferase 2
DOCK10: Dedicator of cytokinesis 10
DYSF: Dysferlin
EPB42: Erythrocyte membrane protein band 4.2
EVL: Enah/Vasp-like
F: Female subjects
F5: Coagulation factor V
FCAR: Fc fragment of IgA receptor
FDR: False discovery rate
FECH: Ferrochelatase
FGF: Fibroblast growth factor
FLOT2: Flotillin 2
FYN: FYN proto-oncogene, Src family tyrosine kinase
GAB2: GRB2 associated binding protein 2
GCCN: GC correction
G-CSF: Granulocyte-colony stimulating factor
GLS: Glutaminase
GM-CSF: Granulocyte-macrophage colony-stimulating factor
GNAI3: G protein subunit alpha I3
GO: Gene ontology
GSK-3β: Glycogen synthase kinase 3 beta
HBQ1: Hemoglobin subunit theta 1
HGF: Hepatocyte growth factor
HK1: Hexokinase 1
HTA: Human transcriptome array
IARS: Isoleucyl-tRNA synthetase
ICH: Intracerebral hemorrhage
iCOS: Inducible co-stimulator
iCOSL: Inducible T cell co-stimulator ligand
IFNGR1: Interferon gamma receptor 1
IFN-γ: Interferon gamma
IGF: Insulin-like growth factor
IL-1: Interleukin 10
IL-10: Interleukin 10
IL17RA: Interleukin 17 receptor A
IL1R1: Interleukin 1 receptor type 1
IL1R2: Interleukin 1 receptor type 2
IL1RAP: Interleukin 1 receptor accessory protein
IL-27: Interleukin 27
IL-6: Interleukin 6
IL6R: Interleukin 6 receptor
IL-8: Interleukin 6
IPA: Ingenuity pathway analysis
IQGAP1: IQ motif containing GTPase activating protein 1
IS: Ischemic stroke
ITK: IL2 inducible T cell kinase
KLF1: Kruppel like factor 1
KLHL2: Kelch like family member 2
LCK: LCK proto-oncogene, Src family tyrosine kinase
LIMK2: LIM domain kinase 2
LRP10: LDL receptor-related protein 10
M: Male subjects
MAML2: Mastermind like transcriptional coactivator 2
MAP K14: Mitogen-activated protein kinase 14
MCL1: MCL1, BCL2 family apoptosis regulator
MED1: Mediator complex subunit 1
MED14: Mediator complex subunit 14
miR3614: MicroRNA 3614
MMP: Matrix metalloproteinase
MMP9: Matrix metalloproteinase 9
MRI: Magnetic resonance imagining
MRPS30: Mitochondrial ribosomal protein S30
mTOR: Mechanistic target of rapamycin kinase
MXD1: MAX dimerization protein 1
NF-κB: Nuclear factor kappa-light-chain-enhancer of activated B cells
NGF: Nerve growth factor
NK: Natural killer
NKT-cells: Natural killer T cells
NLRC4: NLR family CARD domain containing 4
NLRP3: NLR family pyrin domain containing 3
NO: Nitric oxide
NOTCH1: Notch 1
NOTCH3: Notch 3
NRBC: Nucleated red blood cells
NRF2: Nuclear factor, erythroid 2 like 2
NUMB: NUMB, endocytic adaptor protein
OGFOD1: 2-Oxoglutarate and iron-dependent oxygenase domain containing 1
PIK3CD: Phosphatidylinositol-4,5-bisphosphate 3-kinase catalytic subunit delta
PINK1: PTEN-induced putative kinase 1
PIP4K2A: Phosphatidylinositol-5-phosphate 4-kinase type 2 alpha
PKCθ: Protein kinase C theta
PLA2G4A: Phospholipase A2 group IVA
PLBD1: Phospholipase B domain containing 1
PPAR: Peroxisome proliferator-activated receptor
PPARγ: Peroxisome proliferator activated receptor gamma
PYGL: Glycogen phosphorylase L
RASGRP1: RAS guanyl releasing protein 1
REML: Restricted maximum likelihood
RNF123: Ring finger protein 123
ROCK1: Rho associated coiled-coil containing Protein kinase 1
ROS: Reactive oxygen species
rtPA: Recombinant tissue plasminogen activator
S100A8: S100 calcium binding protein A8
S1PR1: Sphingosine-1 phosphate receptor 1
SCL2A1: Solute carrier family 2 member 1
SEPT1: Septin 1
SFK: Src family kinases
SKAP1: Src kinase associated phosphoprotein 1
SLC4A1: Solute carrier family 4 member 1 (Diego blood group)
SMARCA2: SWI/SNF related, matrix associated, actin dependent regulator of chromatin, subfamily A, member 2
SMARCC1: SWI/SNF related, matrix associated, actin dependent regulator of chromatin subfamily C member 1
SMARCC2: SWI/SNF related, matrix associated, actin dependent regulator of chromatin subfamily C member 2
SNRNP200: Small nuclear ribonucleoprotein U5 subunit 200
SPTB: Spectrin beta, erythrocytic
SST: Single space transformation
STAT3: Signal transducer and activator of transcription 3
STX3: Syntaxin 3
SULT1B1: Sulfotransferase family 1B member 1
SUMO: Small ubiquitin-like modifier
TDP1: Tyrosyl-DNA phosphodiesterase 1
TFRC: Transferrin receptor
TGFBR1: (TGF beta receptor 1)
TGF-β1: Transforming growth factor beta 1
Th1: T helper cell 1
Th2: T helper cell 2
TLR: Toll-like receptor
tPA: Tissue plasminogen activator
TRAJ36: T cell receptor alpha joining 36
TTC3: Tetratricopeptide repeat domain 3
U1 snRNP: U1 spliceosomal RNA
U2 snRNP: U2 spliceosomal RNA
U3 snRNP: U3 spliceosomal RNA
U4 snRNP: U4 spliceosomal RNA
U5 snRNP: U5 spliceosomal RNA
U6 snRNP: U6 spliceosomal RNA
VASP: Vasodilator stimulated phosphoprotein
VEGF: Vascular endothelial growth factor
VPS11: VPS11, CORVET/HOPS core subunit
VRF: Vascular risk factor
WDFY3: WD repeat and FYVE domain containing 3
WGCNA: Weighted correlation network analysis
WIPI2: WD repeat domain, phosphoinositide interacting 2
