# Supplementary material for: Inflammatory, regulatory, and autophagy co-expression modules and hub genes underlie the peripheral immune response to human intracerebral hemorrhage
Source: J Neuroinflammation. 2019 Mar 5;16:56. doi: 10.1186/s12974-019-1433-4 (PMC6399982; doi:10.1186/s12974-019-1433-4)

Figure S1. Soft-thresholding plot to determine β based upon soft-thresholding power (A) and connectivity (B). Used all samples (CTRL+ICH) (n=66).

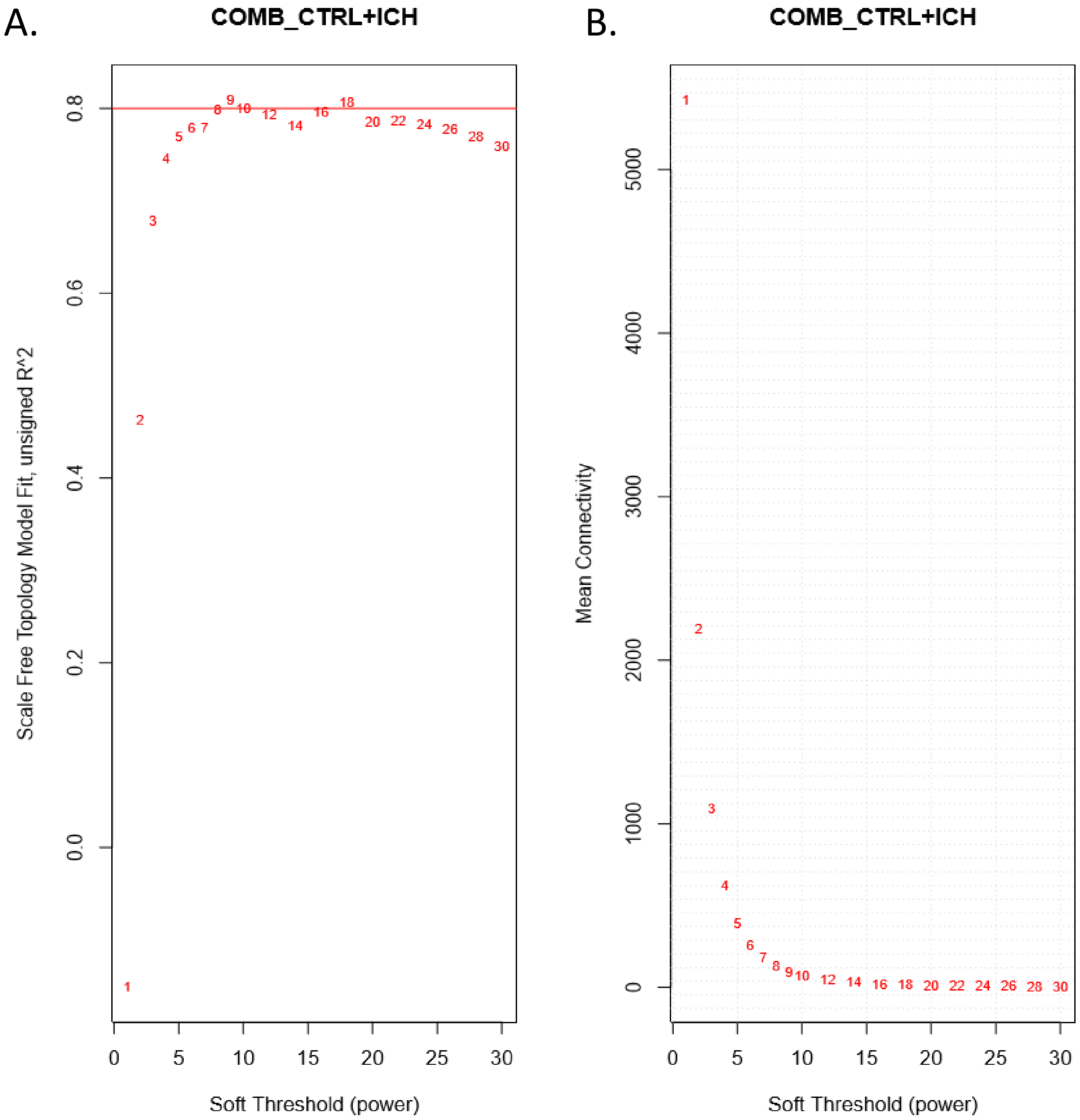

Figure S2. IARS network in the Magenta module. Genes colored in magenta are hub genes.

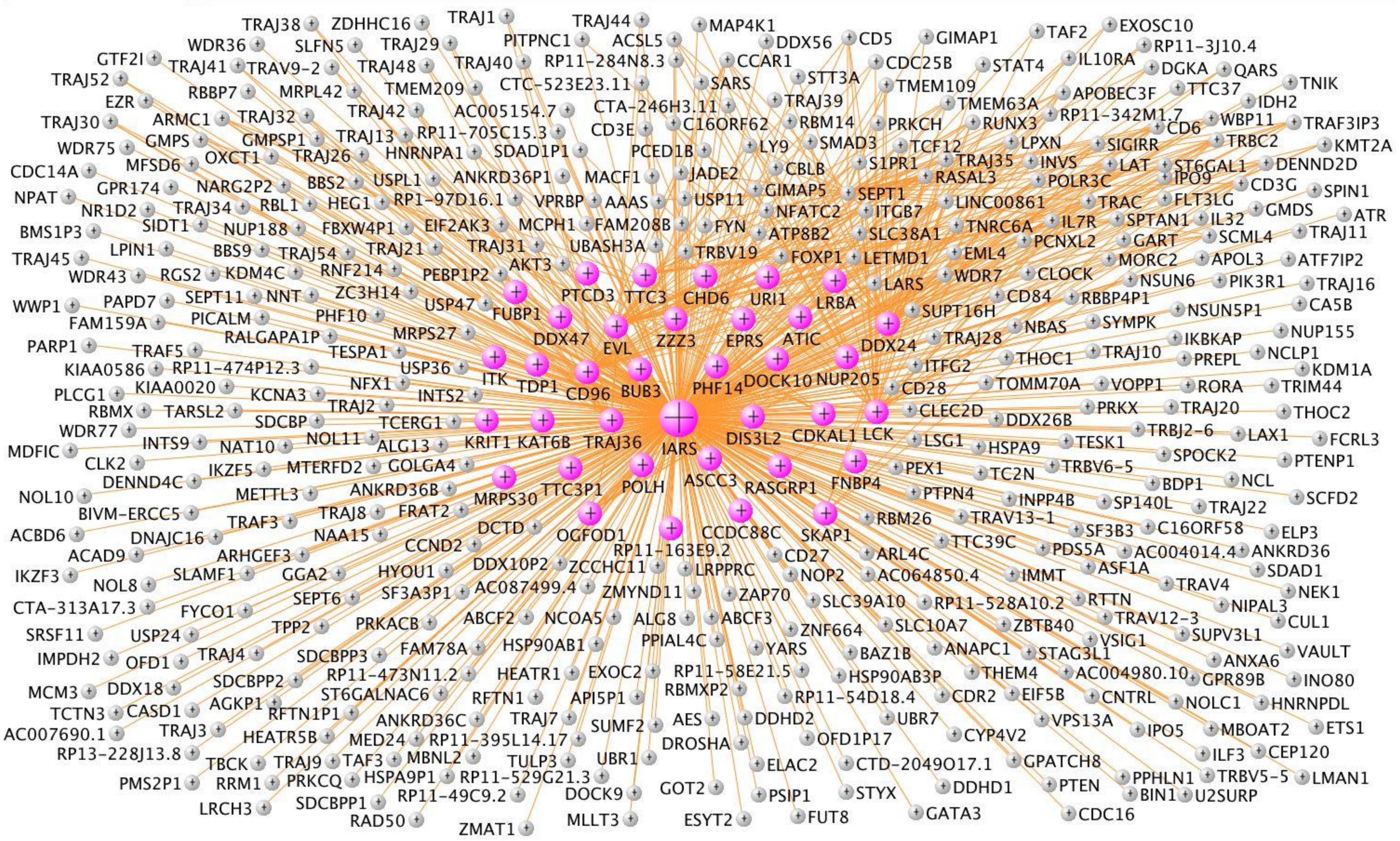

Figure S3. S1PR1 network within the Magenta module. Genes colored in magenta are hub genes.
Note all genes in the S1PR1's network are hub genes.

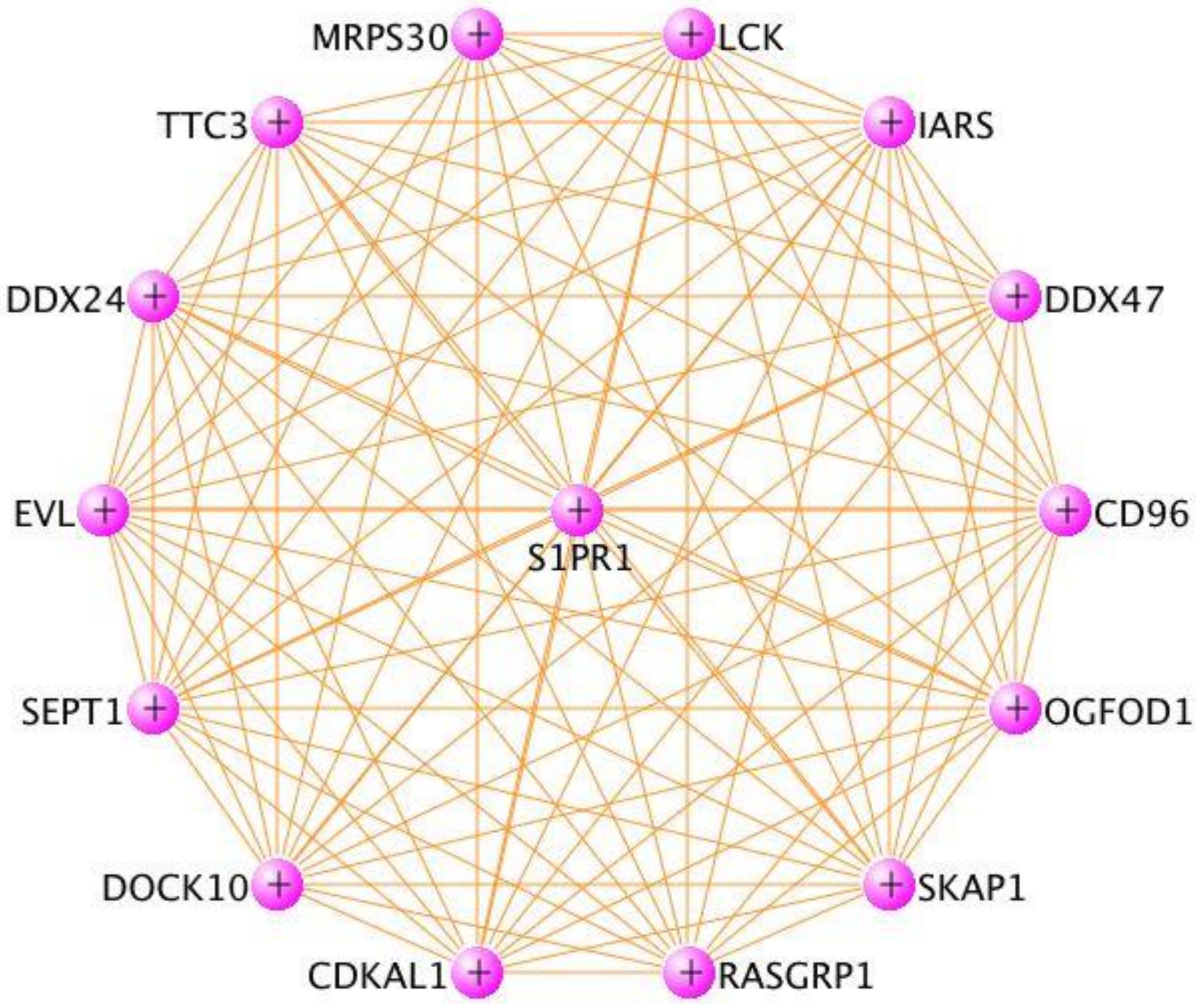

Figure S4. ITK network within the Magenta module. Genes colored in magenta are hub genes.

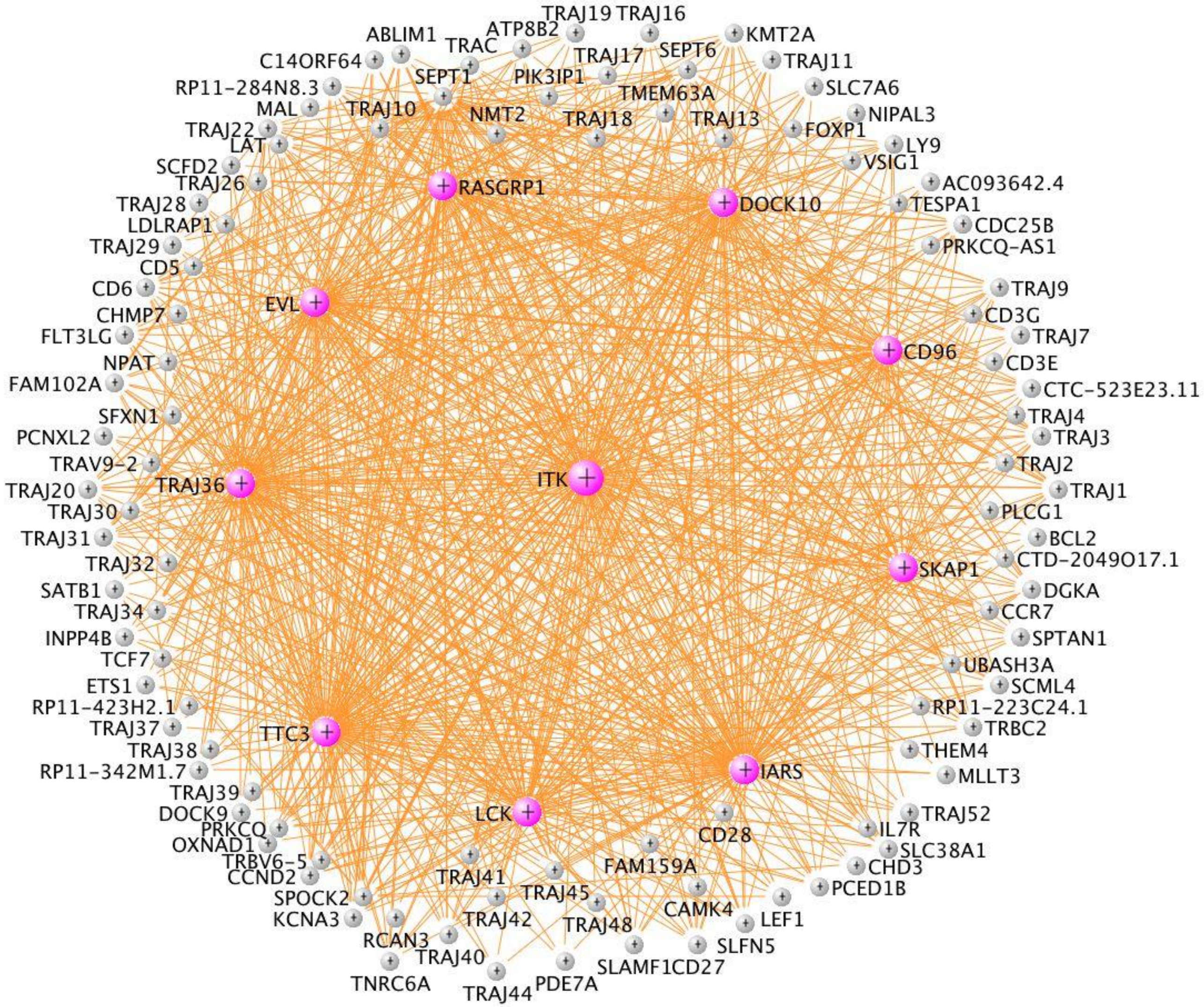

Figure S5. mTOR Network in the Red Module. Genes colored in red are hub genes.

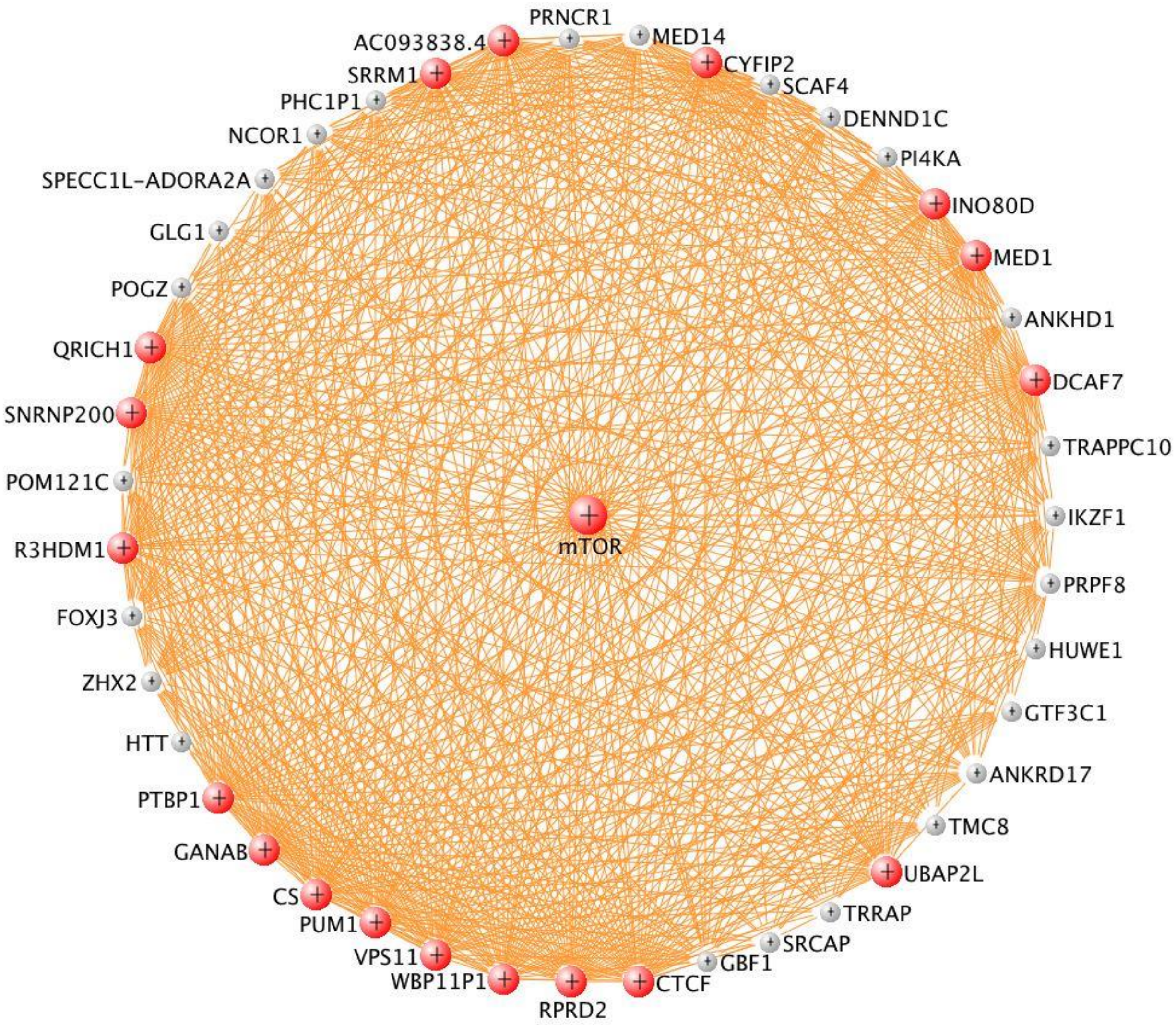

Figure S6. AMBRA1 Network in the Red Module. Genes colored in red are hub genes.

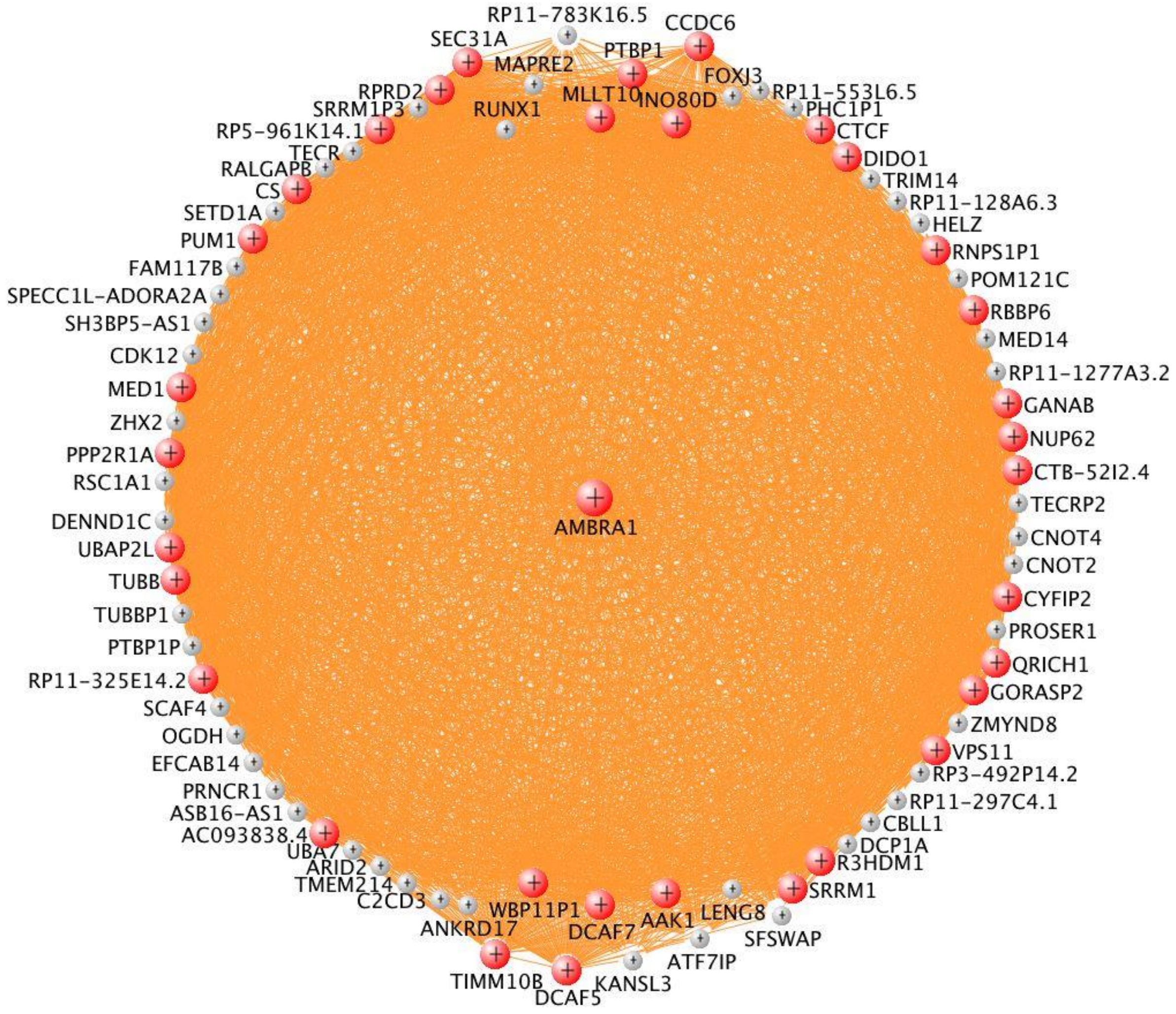

Figure S7. NLRC4 Network in the GreenYellow Module. Genes colored in green-yellow are hub genes.

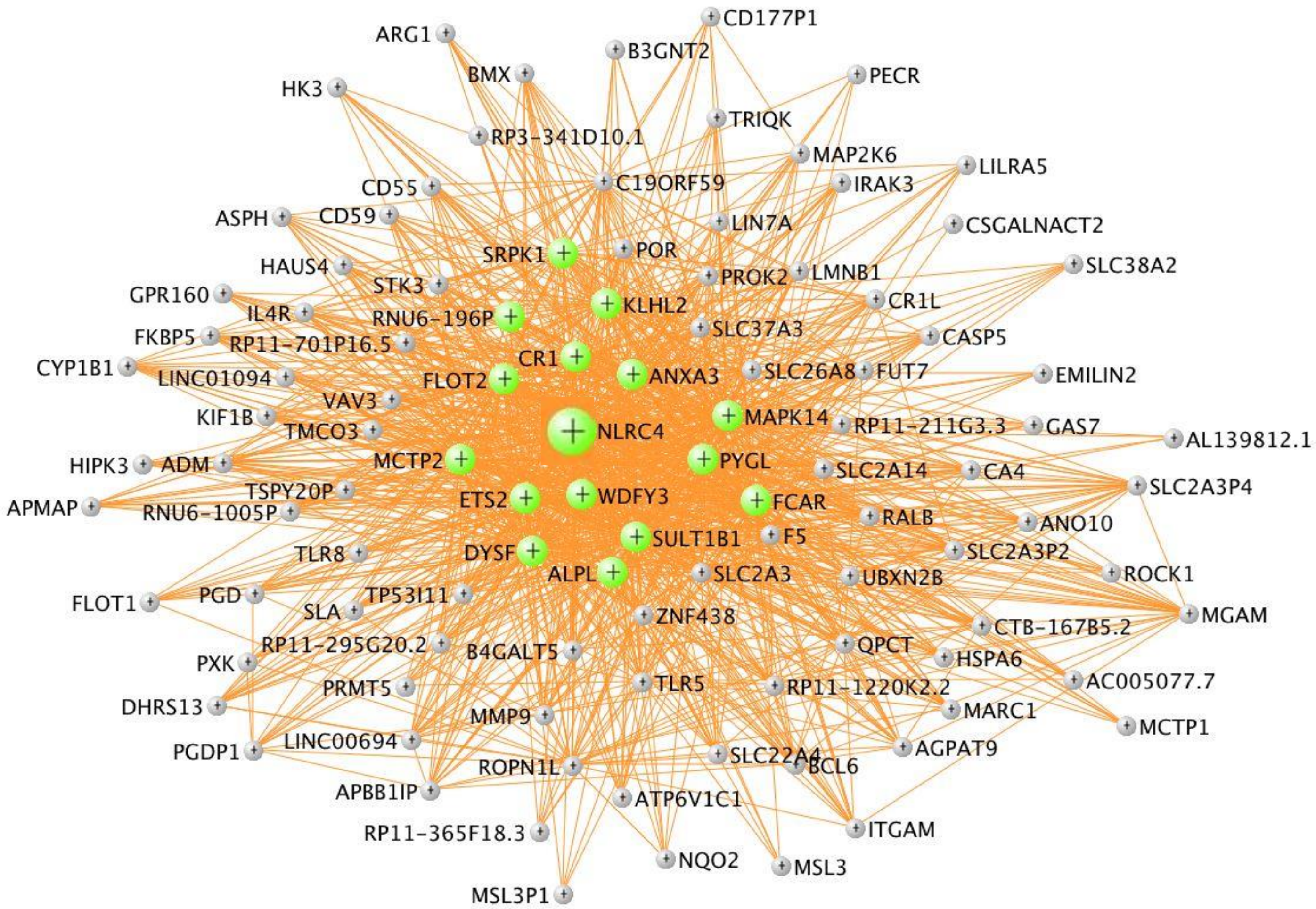

Figure S8. WDFY3 Network in the GreenYellow Module. Genes colored in green-yellow are hub genes.

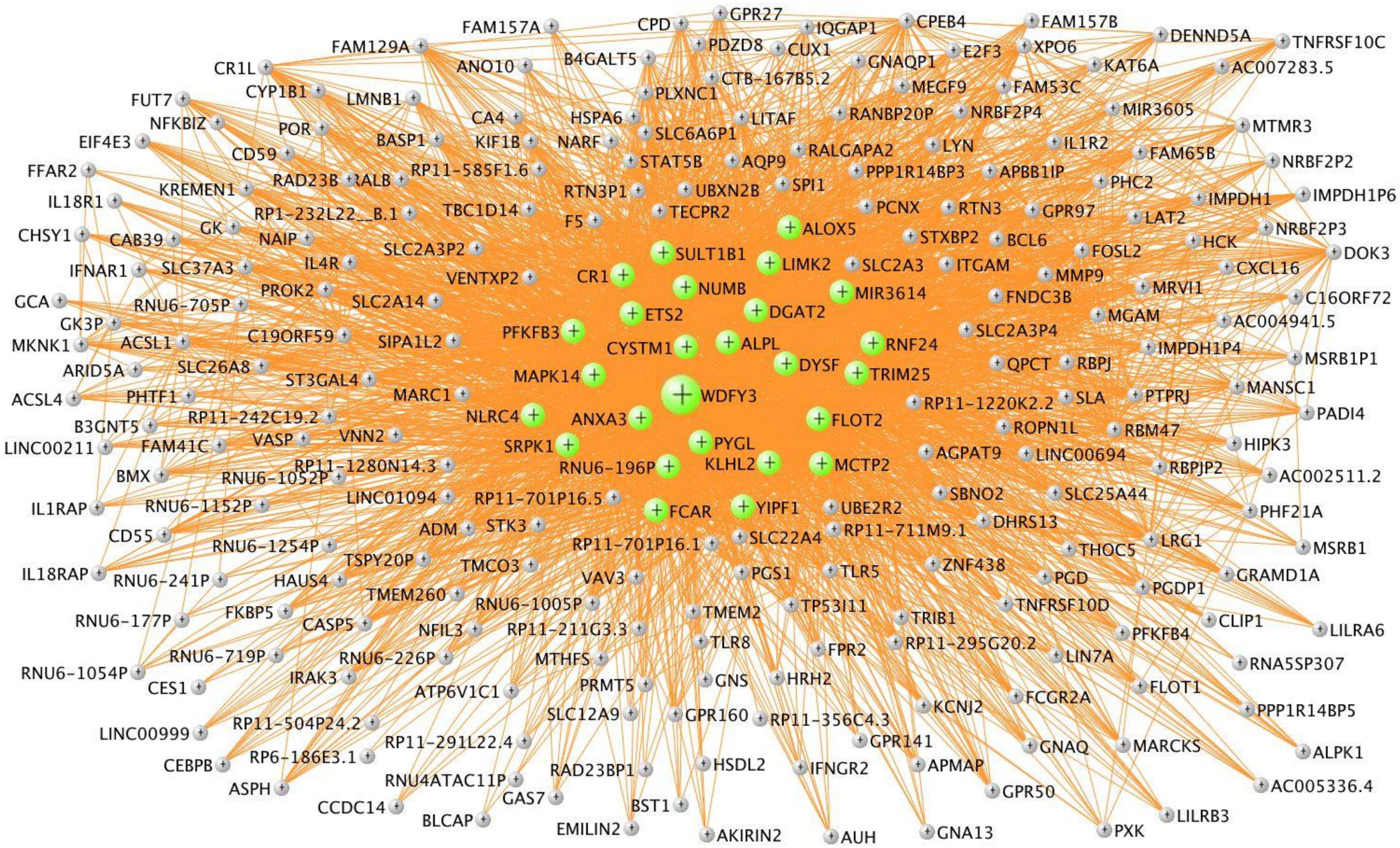

Figure S9. miR3614 Network in the GreenYellow Module. Genes colored in green-yellow are hub genes.

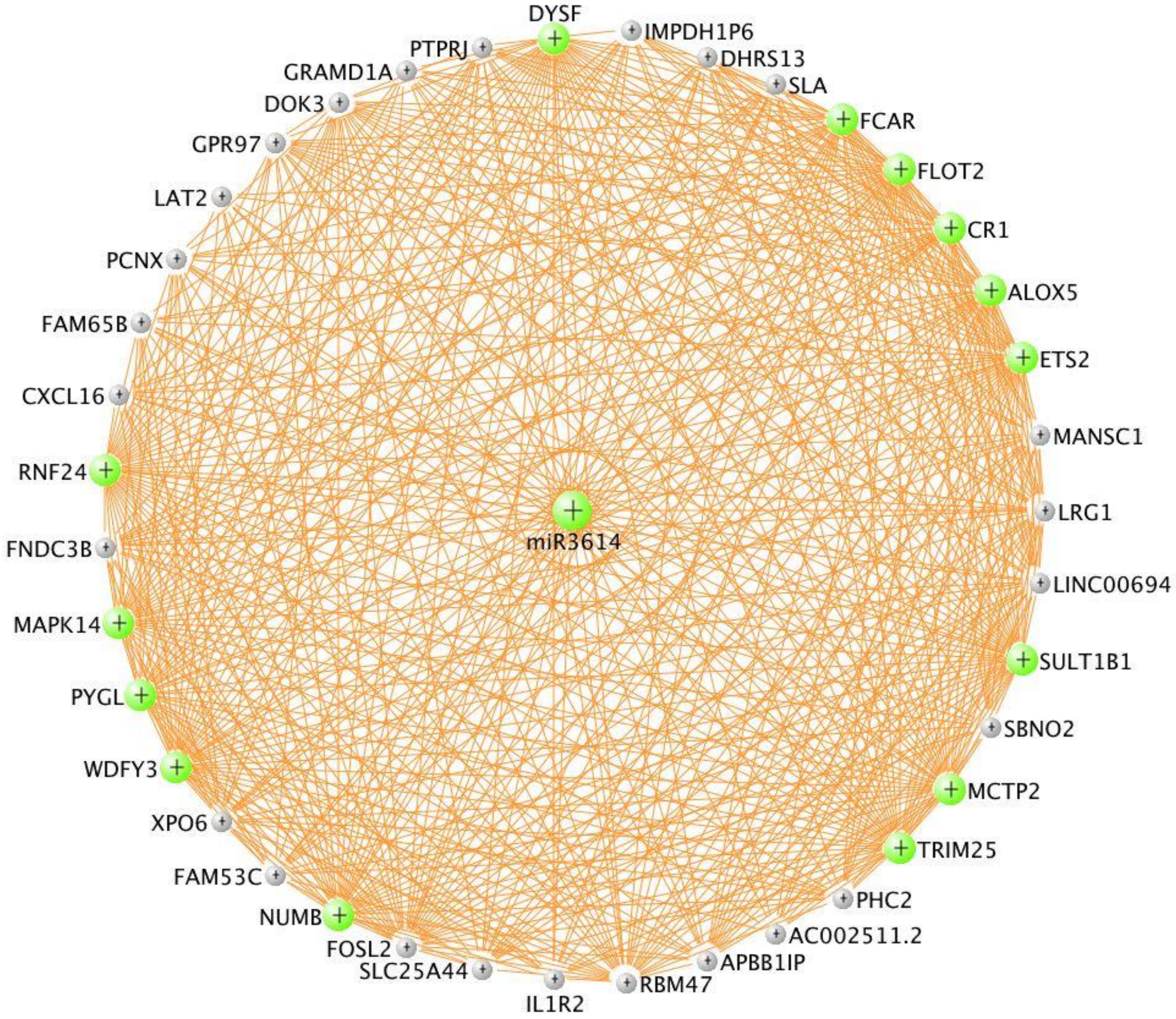

Figure S10. ATG3 Network in the DarkOliveGreen Module. Genes colored in dark green are hub genes.

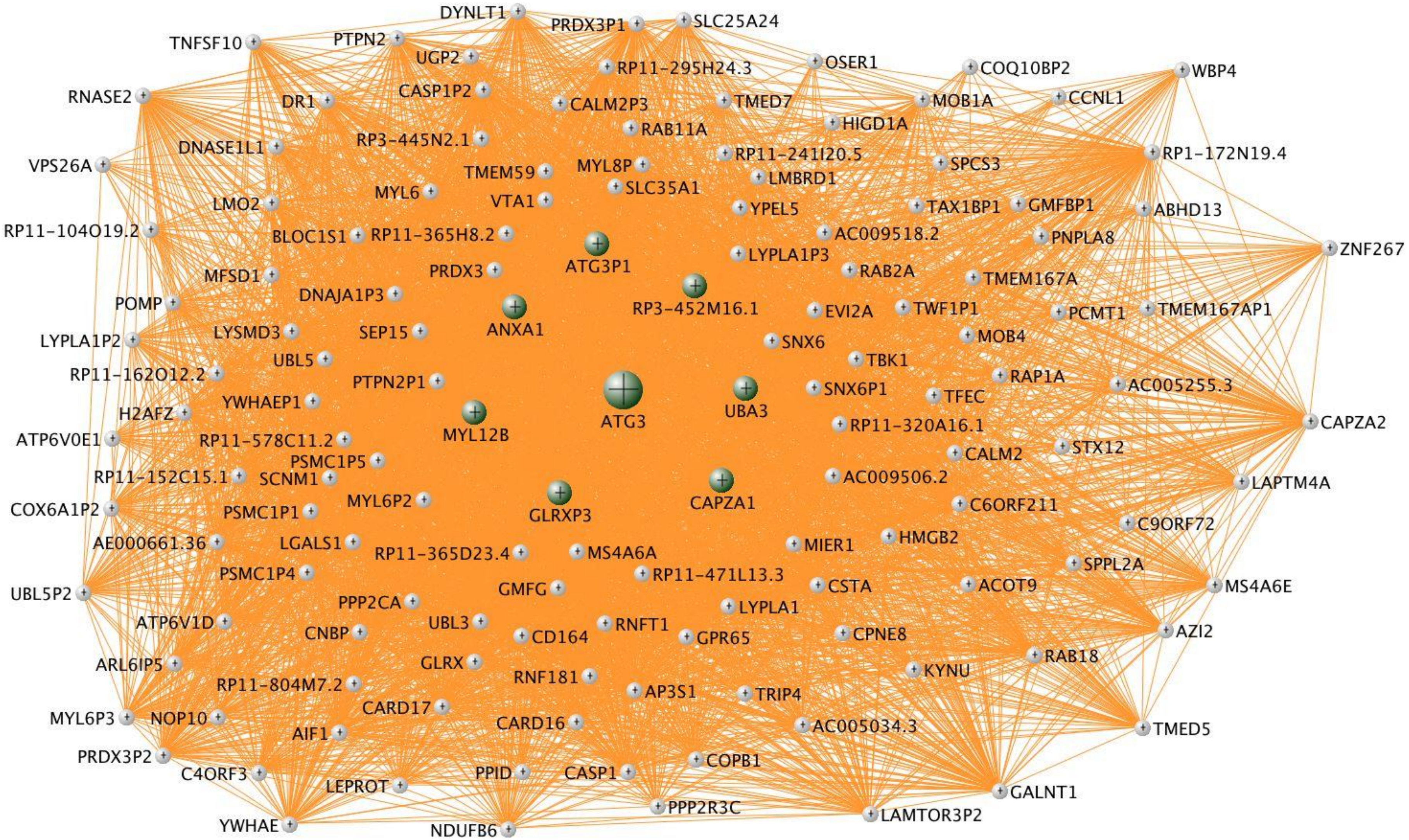

Figure S11. SLC4A1 Network in the Sienna3 Module. Genes colored in brown are hub genes.

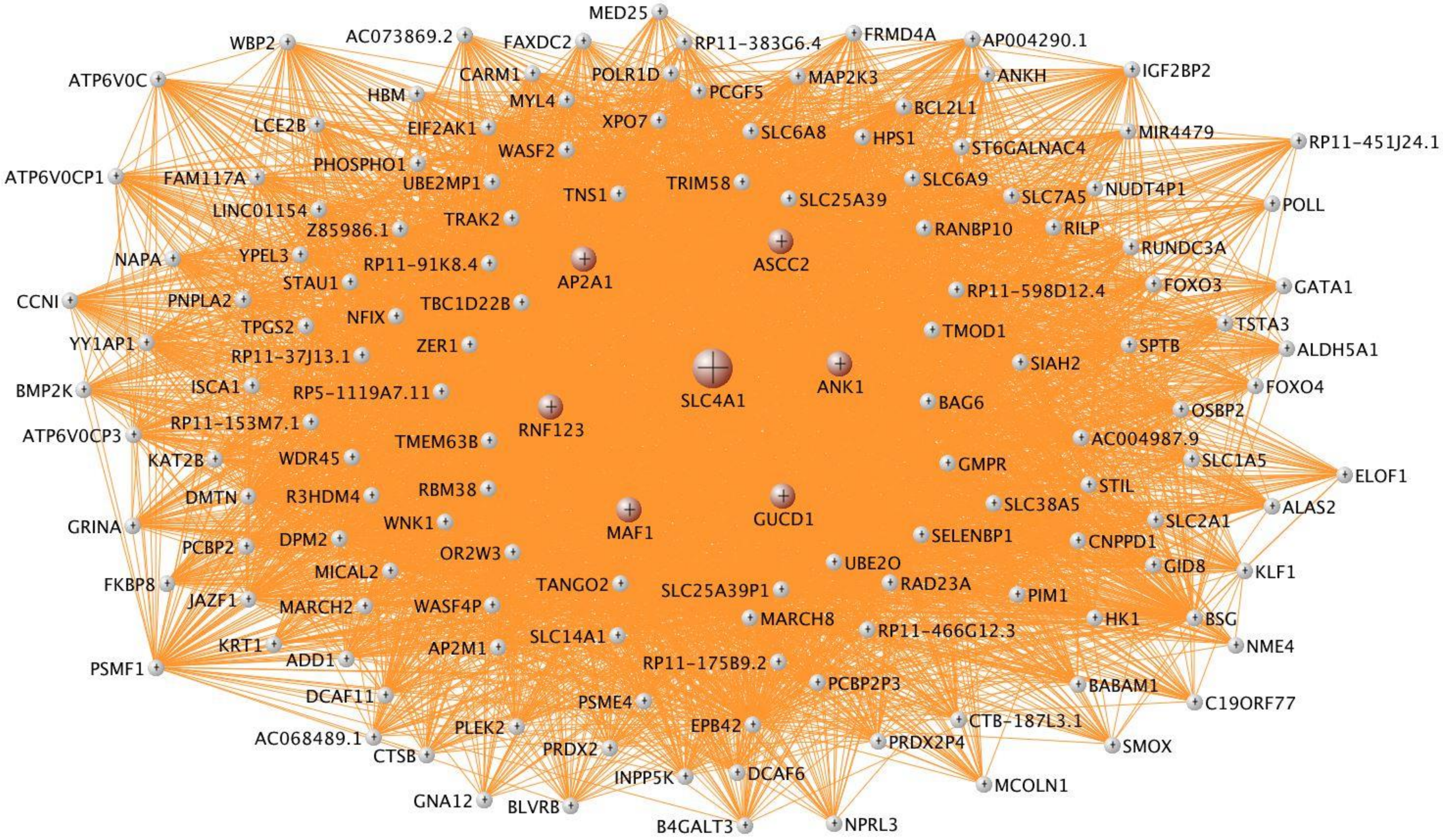

Figure S12. ANK1 Network in the Sienna3 Module. Genes colored in brown are hub genes.

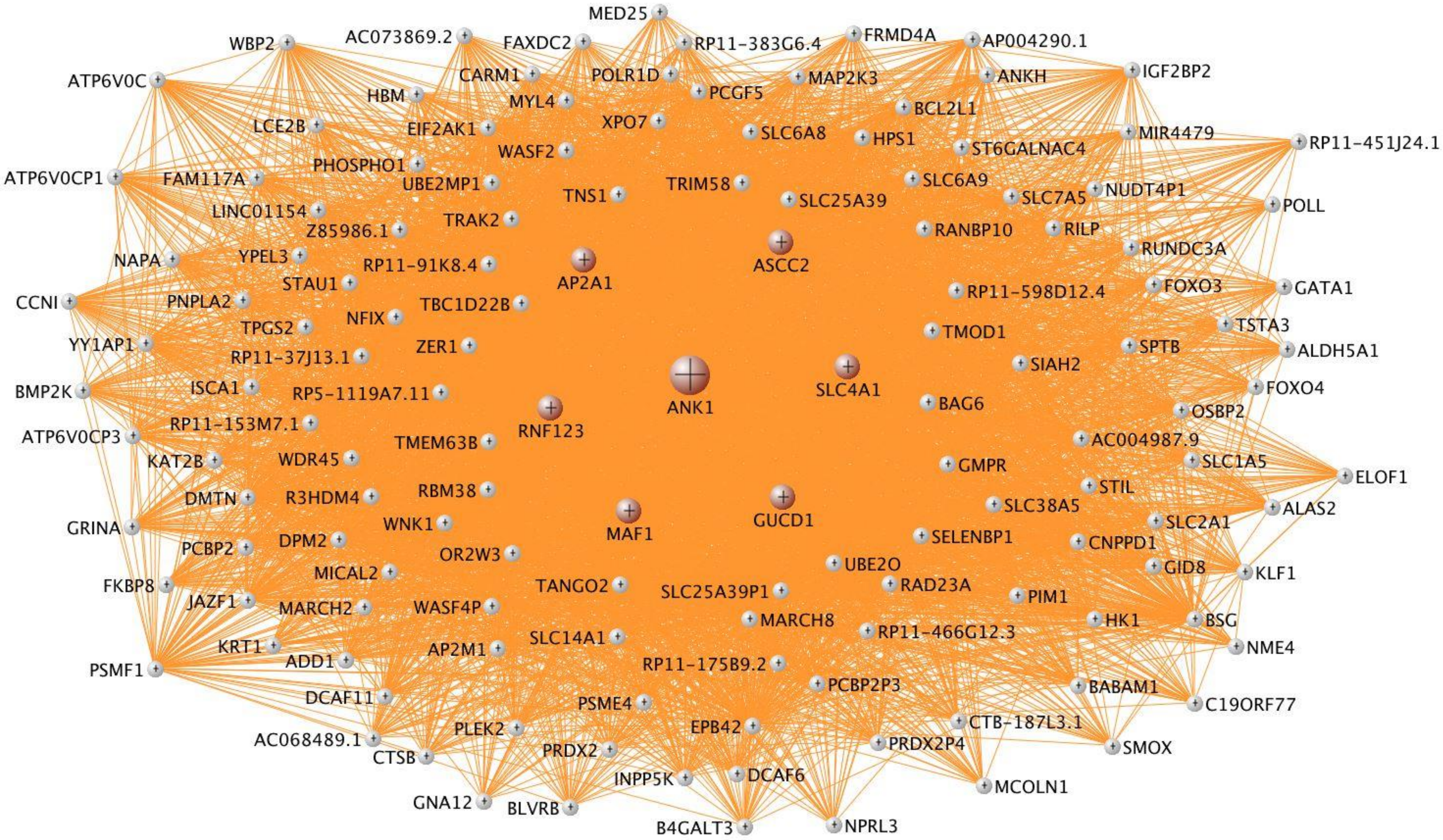

Figure S13. PIP4K2A Network in the Cyan Module. Genes colored in cyan are hub genes.

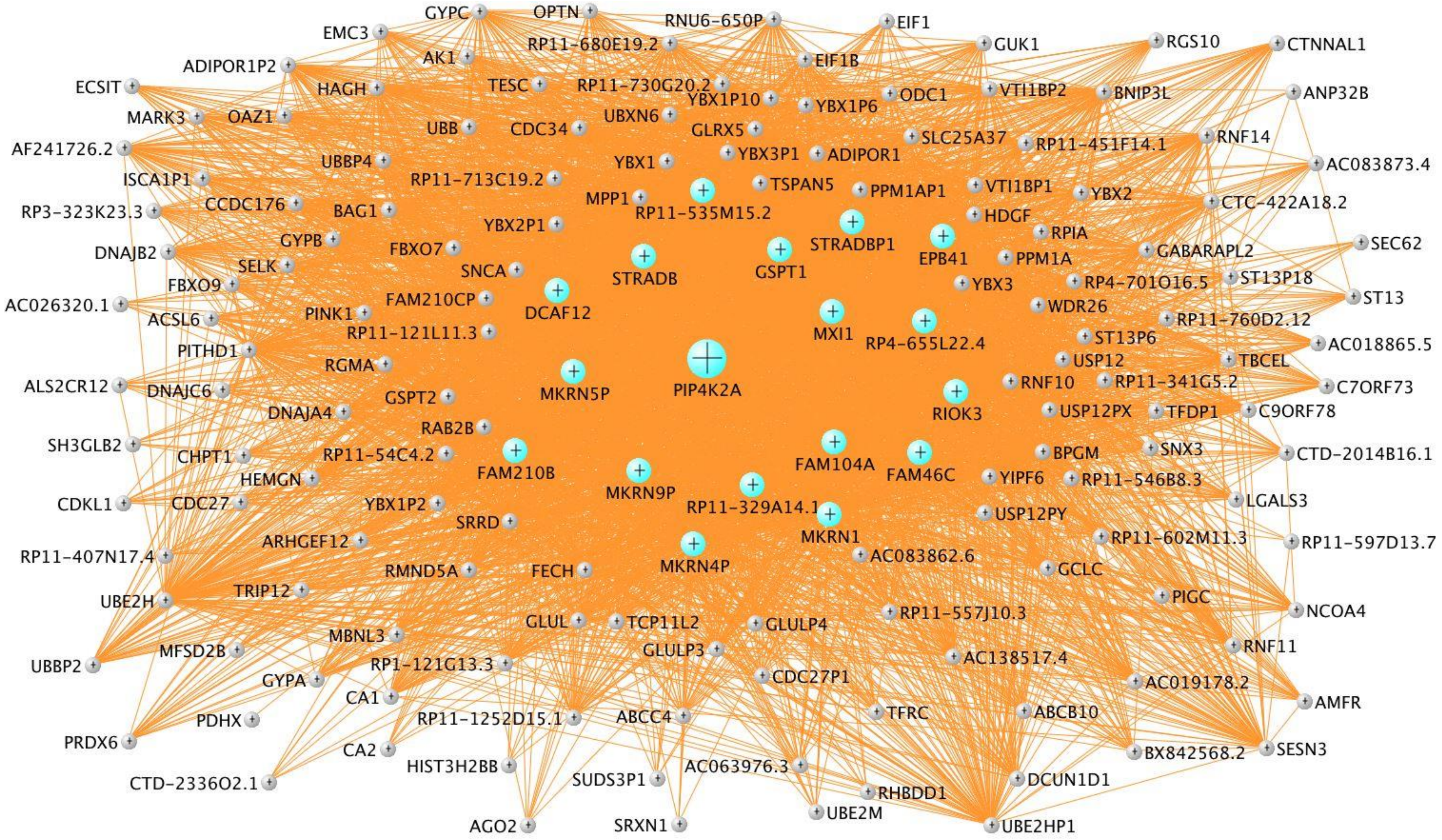

Figure S14. CSF3R Network in the Tan Module. Genes colored in tan are hub genes.

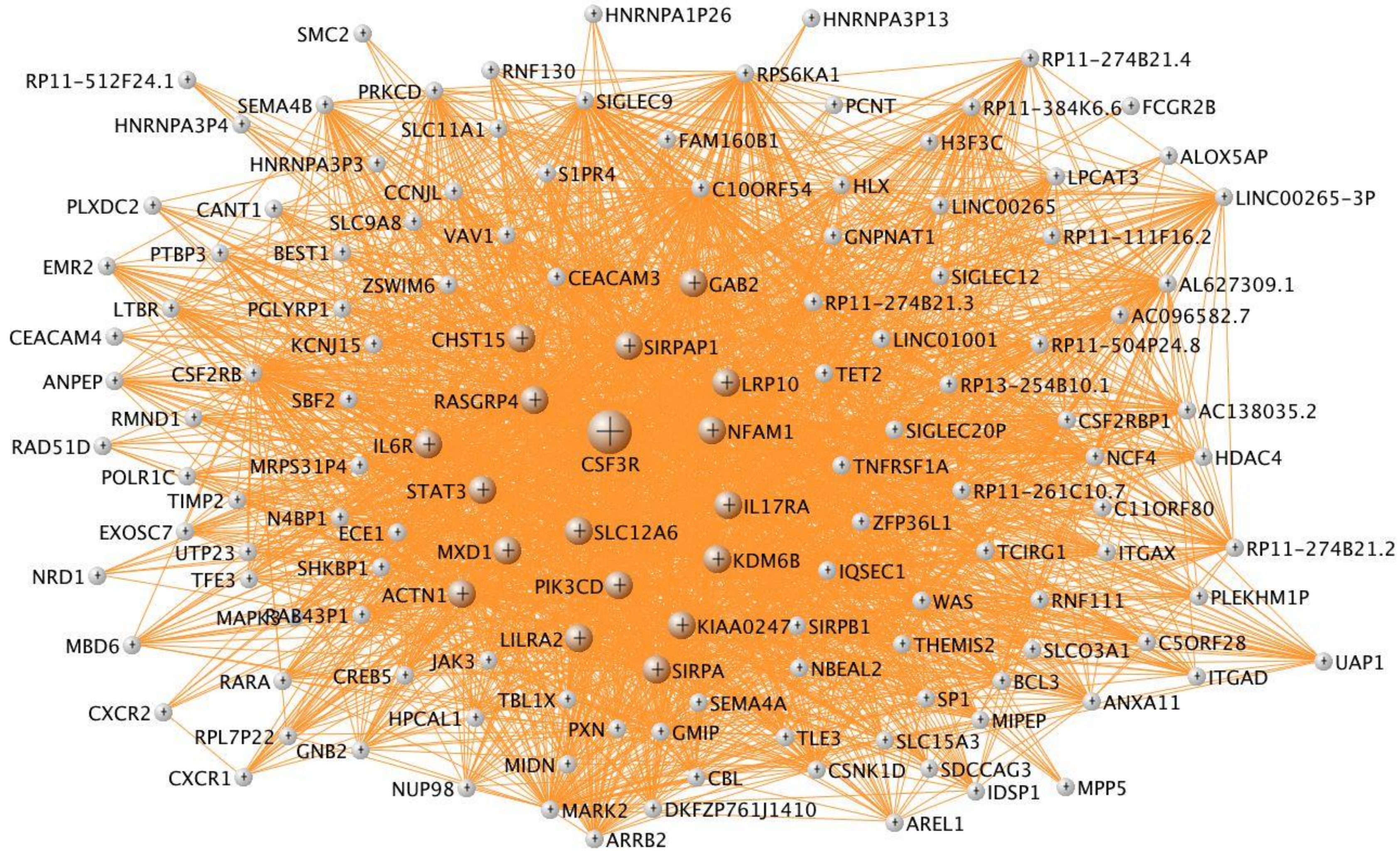

Figure S15. Leukocyte Extravasation Signaling Pathway. Genes circled in purple are present in the Tan Module

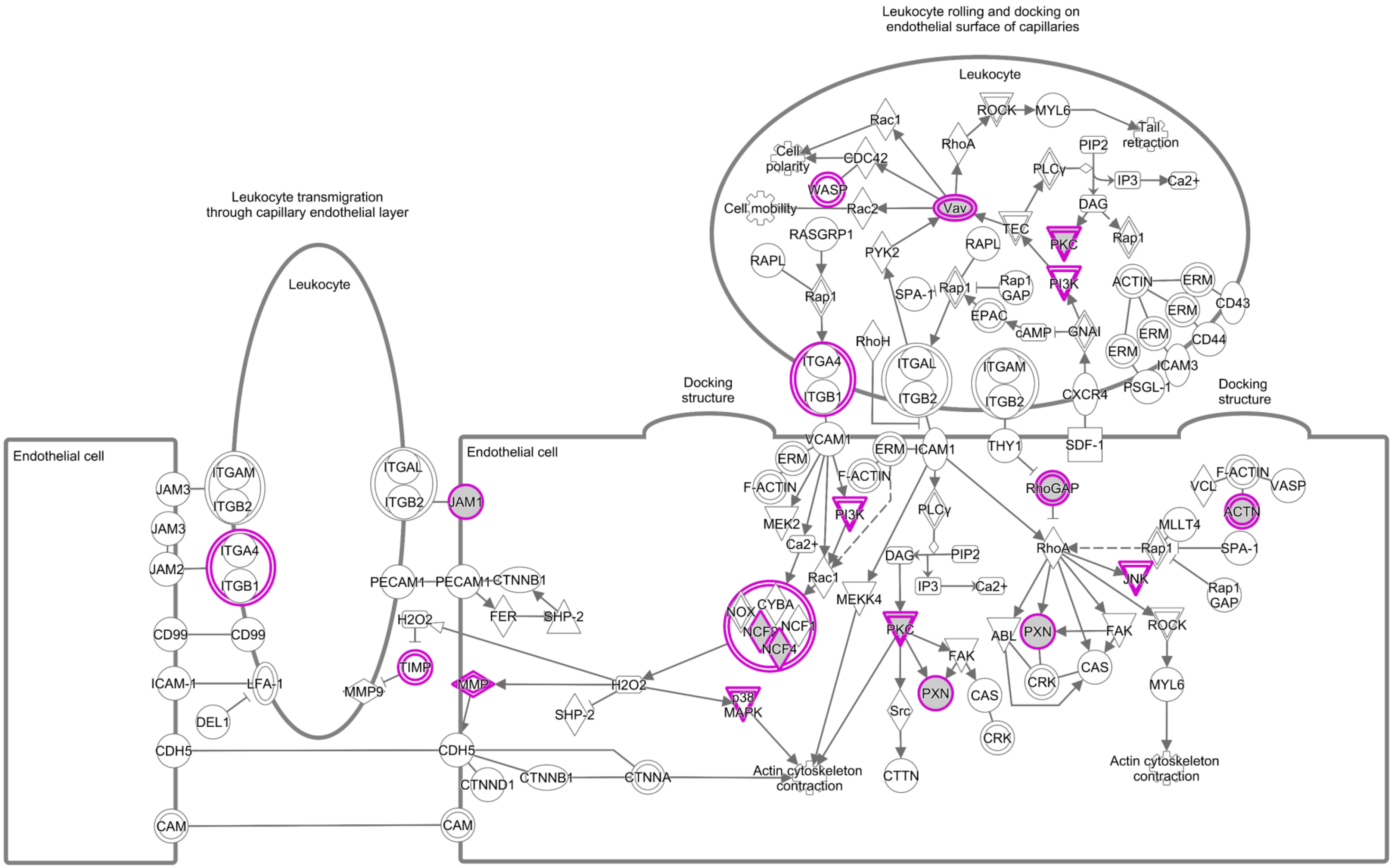

Figure S16. T-cell Receptor Signaling Pathway. Genes circled in purple are present in the Magenta Module.

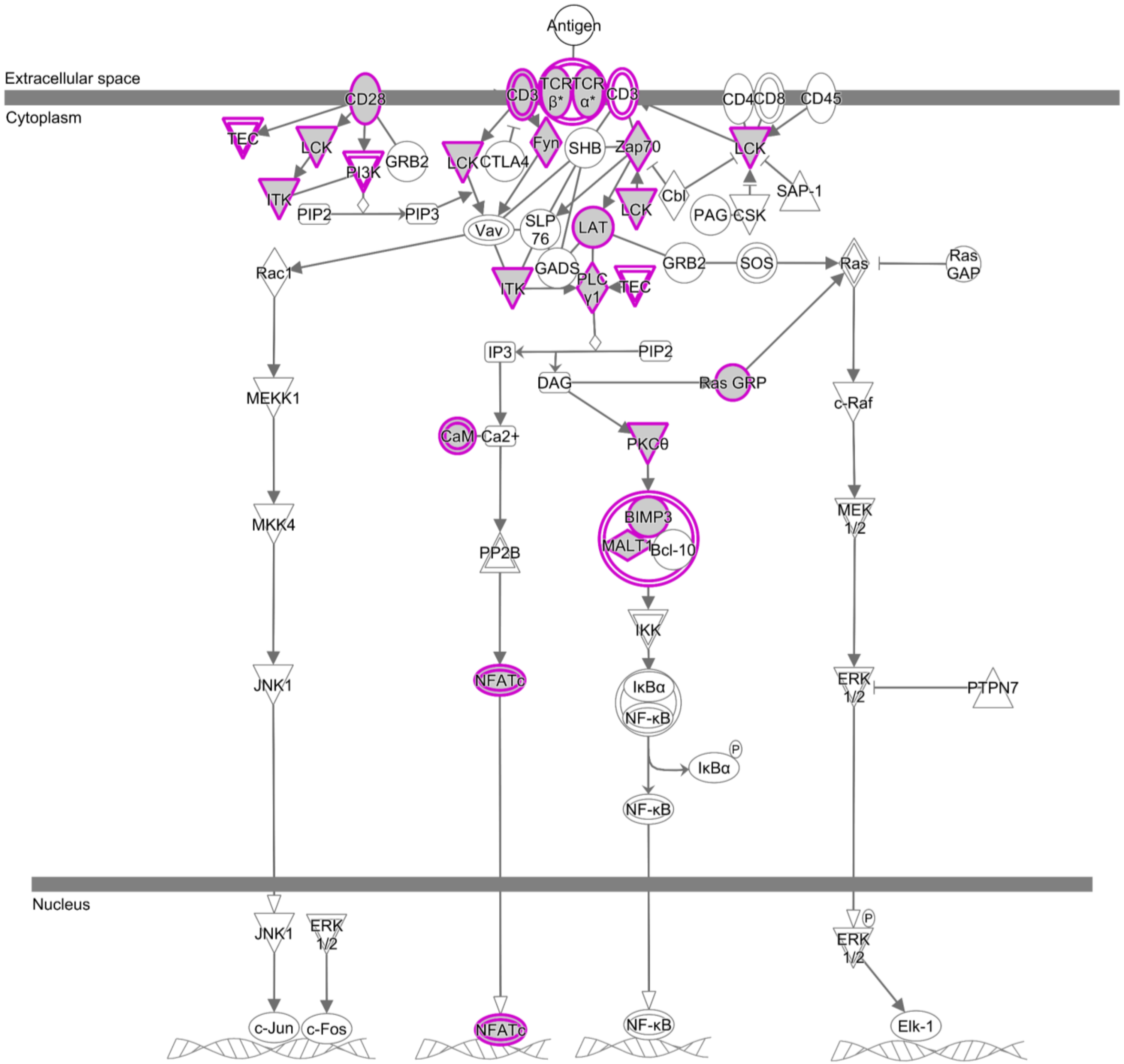

Figure S17. GSK-3β Network in the Tan Module. Genes colored in tan are hub genes.

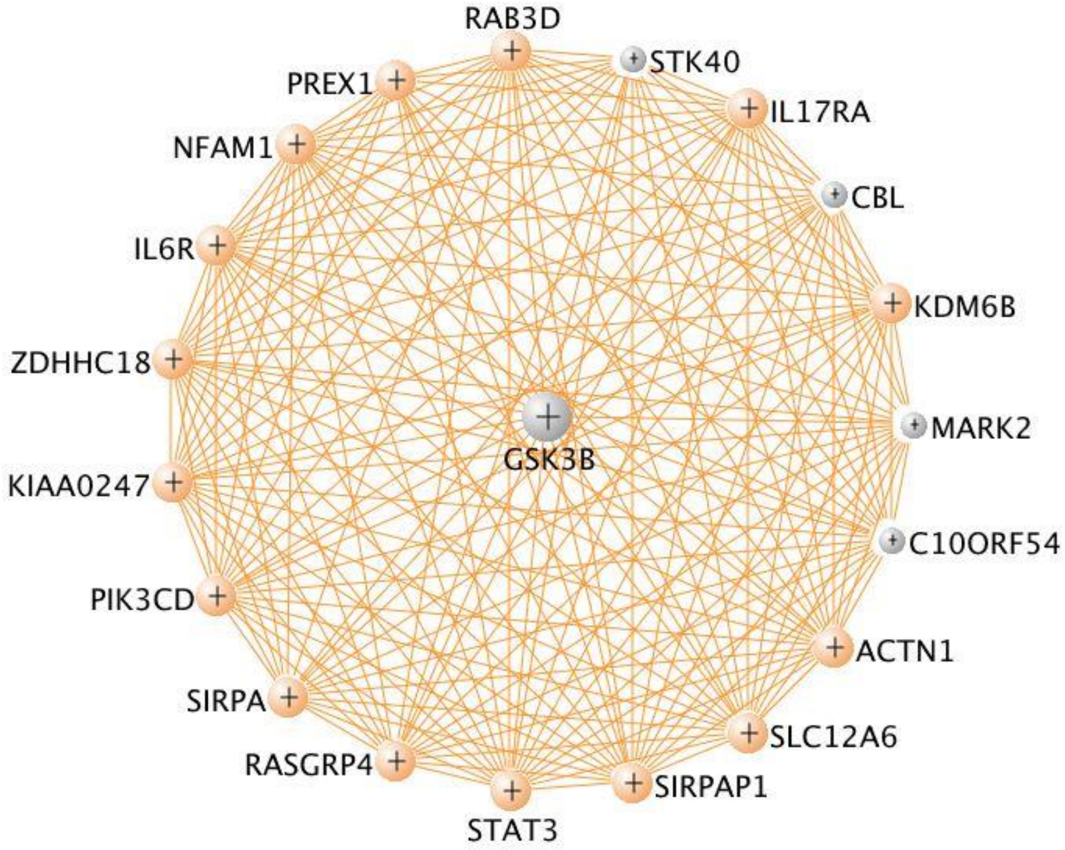

Supplement: Supplementary file 1 — Figure S1. Soft-thresholding power (A) and connectivity (B). Figure S2. IARS network in the Magenta module. Genes colored in magenta are hub genes. Figure S3. S1PR1 network within the Magenta module. Genes colored in magenta are hub genes. Note all genes in the S1PR1’s network are hub genes. Figure S4. ITK network within the Magenta module. Genes colored in magenta are hub genes. Figure S5. mTOR Network in the Red Module. Genes colored in red are hub genes. Figure S6. AMBRA1 Network in the Red Module. Genes colored in red are hub genes. Figure S7. NLRC4 Network in the GreenYellow Module. Genes colored in green-yellow are hub genes. Figure S8. WDFY3 Network in the GreenYellow Module. Genes colored in green-yellow are hub genes. Figure S9. miR3614 Network in the GreenYellow Module. Genes colored in green-yellow are hub genes. Figure S10. ATG3 Network in the DarkOliveGreen Module. Genes colored in dark green are hub genes. Figure S11. SLC4A1 Network in the Sienna3 Module. Genes colored in brown are hub genes. Figure S12. ANK1 Network in the Sienna3 Module. Genes colored in brown are hub genes. Figure S13. PIP4K2A Network in the Cyan Module. Genes colored in cyan are hub genes. Figure S14. CSF3R Network in the Tan Module. Genes colored in tan are hub genes. Figure S15. Leukocyte Extravasation Signaling Pathway. Genes circled in purple are present in the Tan Module. Figure S16. T cell Receptor Signaling Pathway. Genes circled in purple are present in the Magenta Module. Figure S17. GSK-3β Network in the Tan Module. Genes colored in tan are hub genes. (PDF 4696 kb) [file 12974_2019_1433_MOESM1_ESM.pdf]
